# Transcription factor ZEB1 coordinating with NuRD complex to promote oncogenesis through glycolysis in colorectal cancer

**DOI:** 10.3389/fphar.2024.1435269

**Published:** 2024-08-13

**Authors:** Tianyang Gao, Xinhui Hao, Jingyao Zhang, Miaomiao Huo, Ting Hu, Tianyu Ma, Hefen Yu, Xu Teng, Yong Wang, Yunkai Yang, Wei Huang, Yan Wang

**Affiliations:** ^1^ Key Laboratory of Immune Microenvironment and Disease (Ministry of Education), Department of Biochemistry and Molecular Biology, School of Basic Medical Sciences, Tianjin Medical University, Tianjin, China; ^2^ Key Laboratory of Cancer and Microbiome, State Key Laboratory of Molecular Oncology, National Cancer Center/National Clinical Research Center for Cancer/Cancer Hospital, Chinese Academy of Medical Sciences and Peking Union Medical College, Beijing, China; ^3^ Beijing Key Laboratory of Cancer Invasion and Metastasis Research, Department of Biochemistry and Molecular Biology, School of Basic Medical Sciences, Capital Medical University, Beijing, China; ^4^ Department of Ultrasound, National Cancer Center/National Clinical Research Center for Cancer/Cancer Hospital, Chinese Academy of Medical Sciences and Peking Union Medical College, Beijing, China

**Keywords:** WGCNA, Cox regression analysis, ZEB1, the NuRD complex, glycolysis, colorectal cancer

## Abstract

**Background:**

Colorectal cancer (CRC) is an aggressive primary intestinal malignancy with the third-highest incidence and second-highest mortality among all cancer types worldwide. Transcription factors (TFs) regulate cell development and differentiation owing to their ability to recognize specific DNA sequences upstream of genes. Numerous studies have demonstrated a strong correlation between TFs, the etiology of tumors, and therapeutic approaches. Here, we aimed to explore prognosis-related TFs and comprehend their carcinogenic mechanisms, thereby offering novel insights into the diagnosis and management of CRC.

**Materials and Methods:**

Differentially expressed TFs between CRC and normal tissues were identified leveraging The Cancer Genome Atlas database, Weighted correlation network analysis and Cox regression analysis were performed to identify prognosis-related TFs. The cellular functions of hub TF zinc finger E-box binding homeobox 1 (*ZEB1*) were determined using by 5-ethynyl-2′-deoxyuridine and cell invasion assays in CRC cells. RNA-sequencing, Kyoto Encyclopedia of Genes and Genomes enrichment, and gene set enrichment analyses were used to identify the cellular processes in which ZEB1 participates. Immunoaffinity purification, silver staining mass spectrometry, and a chromatin immunoprecipitation assay were conducted to search for proteins that might interact with ZEB1 and the target genes they jointly regulate.

**Results:**

Thirteen central TFs related to prognosis were identified through bioinformatics analysis techniques. Among these TFs, *ZEB1* emerged as the TF most closely associated with CRC, as determined through a combination of regulatory network diagrams, survival curves, and phenotype analyses. ZEB1 promotes CRC cell growth by recruiting the NuRD(MTA1) complex, and the ZEB1/NuRD(MTA1) complex transcriptionally represses glycolysis-associated tumor suppressor genes.

**Conclusion:**

Our study not only identified a hub biomarker related to CRC prognosis but also revealed the specific molecular mechanisms through which ZEB1 affects cancer progression. These insights provide crucial evidence for the diagnosis of CRC and potential treatment opportunities.

## 1 Introduction

Colorectal cancer (CRC) is a malignant neoplasm originating in the colon or rectum and constitutes roughly 10% of all reported cancer cases and associated deaths worldwide, with an estimated annual incidence of around 900,000 cases (Dekker et al., 2019; Guo et al., 2023). Most CRC cases occur sporadically and are primarily linked to modifiable environmental risk factors associated with modern lifestyles, such as obesity, poor diet, alcohol drinking, and smoking (Keum and Giovannucci, 2019; Bai et al., 2022; Yang et al., 2022). The pathological diagnosis of CRC is predominantly reliant on colonoscopy for pathological diagnosis (Brenner et al., 2014; Biller and Schrag, 2021). Treatment for CRC typically involves extensive surgical procedures. However, rectal cancer poses challenges due to its complex anatomy, often leading to a high postoperative recurrence rate (Qin et al., 2023). In contrast, colon cancer tends to have a poor survival prognosis due to its rapid occurrence and metastasis (Compton, 2003; Mao et al., 2024).

Over recent decades, the rapid development of new technologies has enabled us to rapidly obtain extensive physiological and pathological insights into CRC. Weighted gene co-expression network analysis (WGCNA) has emerged as a robust methodology for investigating the intricate associations between genes and phenotypes across various contexts (Esposti et al., 2016; Guo et al., 2017; Liu et al., 2017). A notable advantage of WGCNA lies in its ability to transform gene expression data into coherent co-expression modules, thereby unraveling underlying signaling networks that potentially underpin the observed phenotypic traits. This approach not only facilitates comparative analysis of differentially expressed genes but also elucidates gene interactions within distinct co-expression modules (Wan et al., 2018).

Transcription factors (TFs) are regulatory proteins that govern gene transcription by selectively binding to DNA sequences located upstream of target genes. Therefore, TFs play key roles in developmental processes and differentiation (Lambert et al., 2018; Ulz et al., 2019) and often act as master regulators affecting cell-type decisions (Hainer et al., 2019), ontogeny patterns, and the pathway regulation of many pathways, including the immune response. Numerous studies have linked TFs to tumor development and treatment responses (Krebs et al., 2017; Harley et al., 2018). Consequently, the direct use of CRC-related TFs to construct a prognostic model for cancer holds promise for innovative CRC diagnosis and treatment strategies. Zinc finger E-box binding homeobox 1 (ZEB1), a critical determinant of cellular destiny, tumor initiation, cancer cell adaptability, and metastatic spread across various malignancies (Mohammadi Ghahhari et al., 2022), is considered a transcriptional repressor. It inhibits the transcription of downstream targets, including E-cadherin and miR-200 family members (Burk et al., 2008), by interacting with the promoter regions of these genes. Previous studies have indicated that elevated *ZEB1* expression in CRC promotes invasion and disease progression (Colangelo et al., 2022; Mohammadpour et al., 2022). However, further investigations are needed to ascertain the link between ZEB1 expression and CRC prognosis.

In this study, we employed a WGCNA approach to compare the expression patterns of TFs between patients with and without CRC using data from The Cancer Genome Atlas (TCGA). We developed a prognostic risk model associated with CRC, identifying crucial TFs linked to disease prognosis. Our comprehensive analyses revealed *ZEB1* as a central TF that accelerates tumor progression by promoting cancer cell glycolysis. This finding highlights the significance of investigating the interplay between CRC and glucose metabolism, providing an experimental foundation for future drug development in this field.

## 2 Materials and methods

### 2.1 Data acquisition

The CRC-associated unprocessed RNA-seq and corresponding clinical data were obtained from the UCSC TGCA database (https://portal.gdc.cancer.gov/). Human TFs were obtained from the TRRUST (https://www.grnpedia.org/trust/downloadnetwo-rk.php) and JASPAR (http://jaspar.genereg.net/) database.

### 2.2 WGCNA analysis of differential transcription factors

WGCNA was performed as described previously (Yin et al., 2020). Briefly, network construction and module identification involved four steps: the calculation of topological overlap to determine gene similarity, the generation of a gene clustering tree, the grouping of genes with similar expression into modules, the merging of comparable modules, and the assessment of correlations between different modules and phenotypes. The “WGCNA” R package (https://cran.r-project.org/web/packages/WGCNA/index.html) (Zhang and Horvath, 2005; Langfelder and Horvath, 2008) was utilized to conduct the co-expression network analysis of differentially expressed TFs. The subsequent analysis focused on genes within the module exhibiting the strongest correlation with prognostic traits.

### 2.3 Cox regression model construction

The hub TFs in cancer samples were integrated with survival data, excluding samples without survival data, for batch Cox single-factor regression analysis, using R packages “Survival” (https://cran.r-project.org/web/packages/survival/index.html) and “Survminer” (https://cran.r-project.org/web/packages/survminer/index.html). After regression analysis, the relevant TFs associated with survival were detected by applying a threshold of *p*-value < 0.05 and selected for subsequent least absolute shrinkage and selection operator (LASSO) regression analysis.

Further dimensionality reduction of hub TFs was performed using LASSO regression, and the construction of a risk-scoring model heavily relied on the utilization of the R package “Glmnet” (https://cran.r-project.org/web/packages/glmnet/index.html) (Simon et al., 2011). To enhance the accuracy of our regression model, we initially conducted cross-validation for lambda screening. Subsequently, we selected the model corresponding to lamdba.min and further extracted the expression matrix of genes associated with this model. By utilizing risk score calculations for each sample, the median value was employed as a decisive threshold to categorize samples into high-risk and low-risk groups for subsequent validation procedures. The optimized model was:

risk_score=1.140×ZEB1−0.301×MITF−0.146×APBB1−0.142×CBX7−0.052×HAND2+0.013×LMO3+0.164×KCNIP3+0.189×WWTR1+0.213×MEIS2+0.213×PGR+0.215×NKX3−2+0.311×MEIS1+0.895×TCF7L1.

### 2.4 Cell culture

Human colorectal cancer cell lines (SW480 and HCT116) were obtained from the American Type Culture Collection (ATCC, United States). SW480 cells were cultivated in Dulbecco’s modified Eagle’s medium (DMEM, Biological Industries, Israel) containing 10% fetal bovine serum (FBS) at 5% CO_2_ and 37°C. HCT116 cells were cultivated in Roswell Park Memorial Institute-1640 (RPMI-1640, Biological Industries, Israel) medium containing 10% FBS at 5% CO_2_ and 37°C.

### 2.5 The 5-ethynyl-2′-deoxyuridine (EdU) cell proliferation assay

CRC cells were cultured in 12-well plates and subjected to incubation with an EdU Cell Proliferation Kit (C10310, RiboBio, China) as per the manufacturer’s instructions. The subsequent EdU cell proliferation assay was conducted utilizing a fluorescence microscope, adhering strictly to the provided guidelines.

### 2.6 Cell invasion assays

Matrigel was utilized to coat the Transwell chamber filters. CRC cells transfected with specific siRNAs were seeded into the upper chamber of the Transwell plate and suspended in serum-free DMEM. The lower chamber was filled with a medium containing 10% FBS. The cells in the upper well were eliminated by gently swabbing the top surface of the membrane. The membranes were subsequently subjected to staining and the residual cells were quantified, with a total of four high-power fields evaluated for each individual membrane.

### 2.7 Antibodies and reagents

Anti-HDAC1 (34589), anti-HDAC2 (57156), anti-MTA1 (5646), anti-MTA2 (15793), and anti-MBD3 (99169) antibodies were purchased from Cell Signaling Technology. Anti-RbAp48 (11G10) antibody was purchased from Invitrogen. Anti-MTA3 (A2328), anti-CLDN7 (A2305), anti-ANXA7 (A21109) antibodies were purchased from ABclonal. The siRNAs used in this study were purchased from JTSBIO Co., Ltd.

### 2.8 RNA-seq analysis and real-time quantitative reverse transcription-polymerase chain reaction (qRT-PCR)

Total RNA was isolated from the samples using TRIzol reagent (Invitrogen) in accordance with established protocols. For RNA-seq analysis, three biological replicate samples were prepared and sequenced using an Illumina NextSeq 500. Differentially expressed genes (DEGs) were identified based on a fold change of 1.5 and *p*-value < 0.001, followed by cDNA preparation using the Transcriptor First Strand cDNA Synthesis Kit (Roche, Basel, Switzerland) after total RNA extraction for qRT-PCR analysis. The qRT-PCR was conducted using an ABI PRISM 7500 System (Applied Biosystems), with *β*-actin (ACTB) expression serving as an internal control for quantitation via the comparative Ct method (2^−ΔΔCT^). Supplementary Table S1 lists the primers utilized in this study.

### 2.9 The extracellular acidification rate (ECAR) assay

ECAR was measured using a Seahorse XF96 Extracellular Flux Analyzer, according to the manufacturer’s instructions. Cells were plated into the wells of an XF96 cell culture microplate and incubated at 37°C in a CO_2_ incubator for 24 h to ensure attachment. The assay was initiated after cells were equilibrated for 1 h in XF assay medium supplemented with 10 mM glucose, 5 mM sodium pyruvate, and 2 mM glutamine in a non-CO_2_ incubator. Substrate-based metabolic assays were performed by injecting 10 mM glucose after starvation in XF DMEM assay medium (pH 7.4; Seahorse Bioscience). For starvation, the culture medium was removed at hourly intervals for a total of 5 h and washed once with phosphate-buffered saline, and then starvation was induced by adding XF DMEM assay media (without glucose, pyruvate, or glutamine). During the starvation process, cells were maintained at 37°C in a CO_2_ incubator. The ATP rate assay involved the sequential injection of 2 μM oligomycin and 0.5 μM rotenone/antimycin A.

### 2.10 Immunopurification and mass spectrometry

HEK293T cells were transfected with a plasmid encoding FLAG-tagged ZEB1 and subsequently collected 48 h post-transfection. Cell lysates obtained from approximately 5 × 10^8^ cells were incubated with an anti-FLAG M2 affinity gel (Sigma) for at least 4 h to facilitate the adsorption of the protein complex onto the column resin. Subsequently, elution of the FLAG protein complex was performed using the FLAG peptide (0.2 mg/mL; Sigma-Aldrich), following the manufacturer’s protocol. The collected proteins were then resolved on a sodium dodecyl sulfate-polyacrylamide gel, subjected to silver staining, and finally analyzed through liquid chromatography-tandem mass spectrometry.

### 2.11 Western blotting

Cells were washed with ice-cold PBS before being added to radio immunoprecipitation assay (RIPA) lysate and were then lysed at 4°C for 10 min. The BCA protein level was quantified, and protein lysate was added to the loading buffer for 10 min at 95°C. Electrophoresis was carried out at 100 V. Polyvinylidene fluoride was used to transfer the proteins from the gel to the membrane. The primary antibody (diluted in primary antibody diluent) was added and incubated overnight at 4°C. The secondary antibody prepared in 5% skimmed milk was added the next day, and the membranes were incubated for 1 h at 25°C with gentle mixing. Chemiluminescence images were obtained using a darkroom development technique.

### 2.12 The glutathione S-transferase (GST) pull-down assay

GST-fused nucleosome remodeling and deacetylase (NuRD) subunit constructs were expressed in BL21 *Escherichia coli*, while ZEB1 was subjected to *in vitro* transcription and translation using a rabbit reticulocyte lysate kit (TNT Systems, Promega). About 5 g of the GST fusion proteins were incubated with 5–8 μL of the in vitro-transcribed/translated products in binding buffer at 37°C for 30 min and supplemented with a protease inhibitor mixture. Following five washes with binding buffer, the resulting mixture was subjected to Western blot analysis.

### 2.13 Chromatin immunoprecipitation (ChIP) and quantitative ChIP (qChIP) assays

ChIP experiments were performed on SW480 cells as previously described (Wang et al., 2009). In brief, a total of 1 × 10^7^ cells were subjected to cross-linking with 1% formaldehyde, followed by sonication and pre-clearance. The cell lysates were incubated with 2 µg of specific antibody. The resulting complexes underwent five rounds of washing using low- and high-salt buffers, after which DNA was purified utilizing a QIAquick PCR Purification Kit. For qRT-PCR analysis, the TransStart Top Green qPCR Supermix (TransGen Biotech, Shanghai, China) was employed. The primer sequences utilized in this study can be found in Supplementary Table S2.

### 2.14 Statistical analysis

All statistical analyses and corresponding visualization were performed using the R Studio software version 3.6.3 (RStudio, United States) and SPSS Statistics software (SPSS, Inc., United States). Statistical data were analyzed by Student’s *t*-test. All experimental data were analyzed and visualized with R Studio or GraphPad Prism 8 (GraphPad Software, Inc, United States). Kaplan-Meier curve analyses were performed using the “survminer” R package. For all statistical tests, a two-tailed *p*-value <0.05 denoted statistical significance, which is indicated by *p-value < 0.05 and **p-value < 0.01.

## 3 Results

### 3.1 Identification of differentially expressed TFs and WGCNA analysis in CRC

**FIGURE 1:**
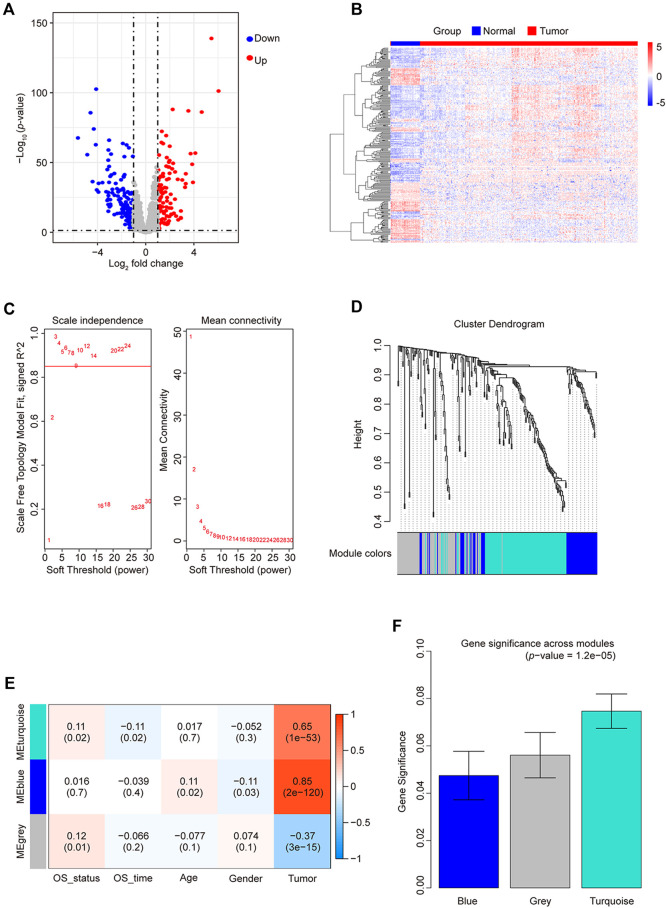
Differentially expressed TF statistics and WGCNA results in CRC. **(A)** Volcano plot displaying differentially expressed TFs. **(B)** Heatmap of differently expressed TFs. **(C)** Analysis of the network topology at various soft-threshold powers. Check scale-free topology; the adjacency matrix was defined using soft thresholds with *β* = 3. **(D)** Clustering dendrograms of TFs with dissimilarity based on topological overlap, together with assigned module colors. **(E)** Calculation of the correlation between the module and the phenotype. OS, overall survival. **(F)** Bar plot indicating mean significance across modules. Gene significance represents the correlation between modules and CRC.

We analyzed the expression matrix of 981 non-redundant human-reported TFs sourced from the JASPAR and TRRUST databases to explore the clinical significance of their dysregulation in CRC development. A total of 430 tissue samples from the TCGA database (379 cancer and 51 adjacent tissues; Table 1) were used to perform differential expression analysis, with the criteria of abs (log_2_ fold change) > 1 and false discovery rate (FDR) < 0.05. From this analysis, we identified 250 differentially expressed genes (DEGs) as CRC-related TFs (Figures 1A, B).

**TABLE 1:** Sample prognostic traits statistics.

	Tumor	Normal
Gender		
Male	208	23
Female	171	28
Age (years)		
Mean	64	69
Median	66	73
Pathologic_stage		
Stage I	57	8
Stage II	137	24
Stage III	114	9
Stage IV	52	9

By employing WGCNA, we constructed a co-expression network using 250 candidate TFs for CRC. We built the network using a one-step method with 3 as the power (R^2^ > 0.85), further merging similar modules with height < 0.25 (Figure 1C), yielding only two module groups: 113 genes in the turquoise module, 62 in blue, and 75 unclassified (gray) (Figure 1D). We evaluated the correlation of TFs from these modules with the prognostic phenotype overall survival status (OS_status). The TFs in the turquoise modules exhibited the highest correlation (correlation coefficient of 0.11; *p*-value = 0.02) with OS_status (Figure 1E). Calculating the correlation matrix between TFs and OS_status in each module generated the gene significance (GS) index (Figure 1F). The TFs in the turquoise module demonstrated the highest average GS index. Therefore, we employed Cytoscape to screen TFs in the turquoise module based on their degree. At a degree of up to 112, 62 TFs were selected with a higher node count. Subsequently, there were none hub TFs when the degree was increased again. Finally, we identified 62 hub TFs (Supplementary Table S3) using a degree threshold of 112 for subsequent analysis.

### 3.2 Construction of prognostic TFs for CRC

**FIGURE 2:**
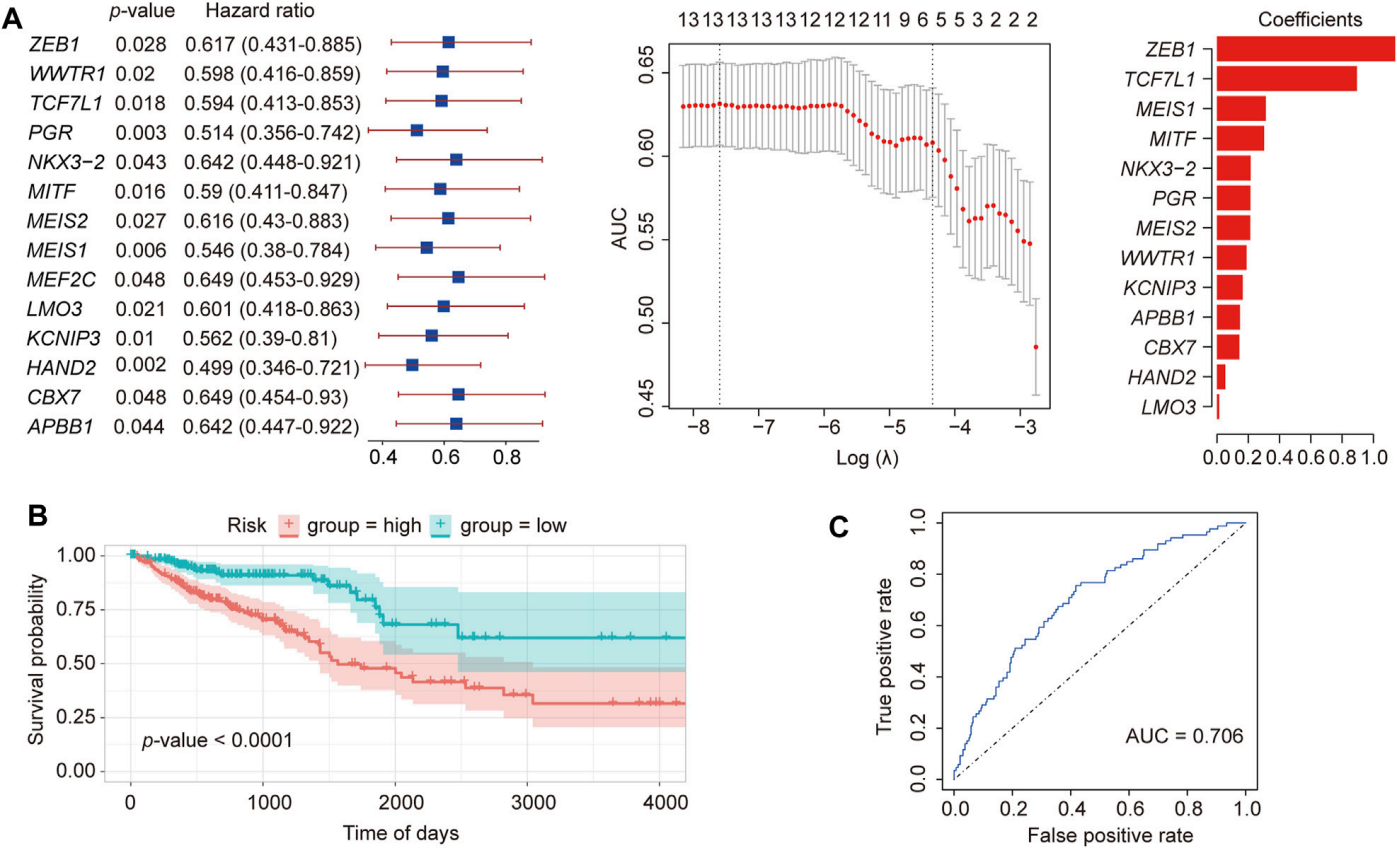
Construction of a prognostic TF model of CRC. **(A)** LASSO regression of 13 differential TFs constructed using Cox single-factor regression. **(B)** Kaplan-Meier curve verification for overall survival and receiver operator characteristics curve. **(C)** Verification of the prognostic model.

To identify key TFs associated with CRC prognosis, 62 hub TFs were analyzed in 378 cancer samples with prognostic data. Through single-variable Cox regression analysis using the “clusterProfiler” R package (http://www.bioconductor.org/packages/release/bioc/html/clusterProfiler.html) (Wu T. et al., 2021), we identified a total of 14 TFs exhibiting a high correlation [hazard ratio < 1, 95% confidence interval (CI) < 1] (Figure 2A). The enrichment analysis results of Gene Ontology (GO) and Kyoto Encyclopedia of Genes and Genomes (KEGG) pathways for these 14 TFs are presented in Supplementary Figure S1. By employing LASSO regression analysis on these 14 TFs (Figure 2A), we cross-validated 13 TFs based on the minimum lambda value, namely, lambda.min, and constructed a cancer-related prognostic risk-scoring model. This model effectively stratified 378 cancer samples into high- and low-risk groups, determined by their respective median scores (189 samples in each group). The Kaplan-Meier curve demonstrated a significant correlation between the high-risk group and unfavorable prognosis in CRC (*p*-value < 0.0001, Figures 2B, C). Moreover, the receiver operating characteristic (ROC) curve demonstrated an impressive area under the curve (AUC) value of 0.706, indicating excellent model performance and enhanced predictive capability.

To validate our model, we also downloaded CRC-related data from the GEO database (GSE14333, Supplementary Figure S2). In the Kaplan-Meier curve of the external dataset, we observed a highly significant association between the high- and low-risk groups and survival time (*p*-value = 0.00082), providing strong evidence for the suitability of our model in prognostic prediction.

### 3.3 Multivariate cox regression analysis and TF-target gene network construction

**FIGURE 3:**
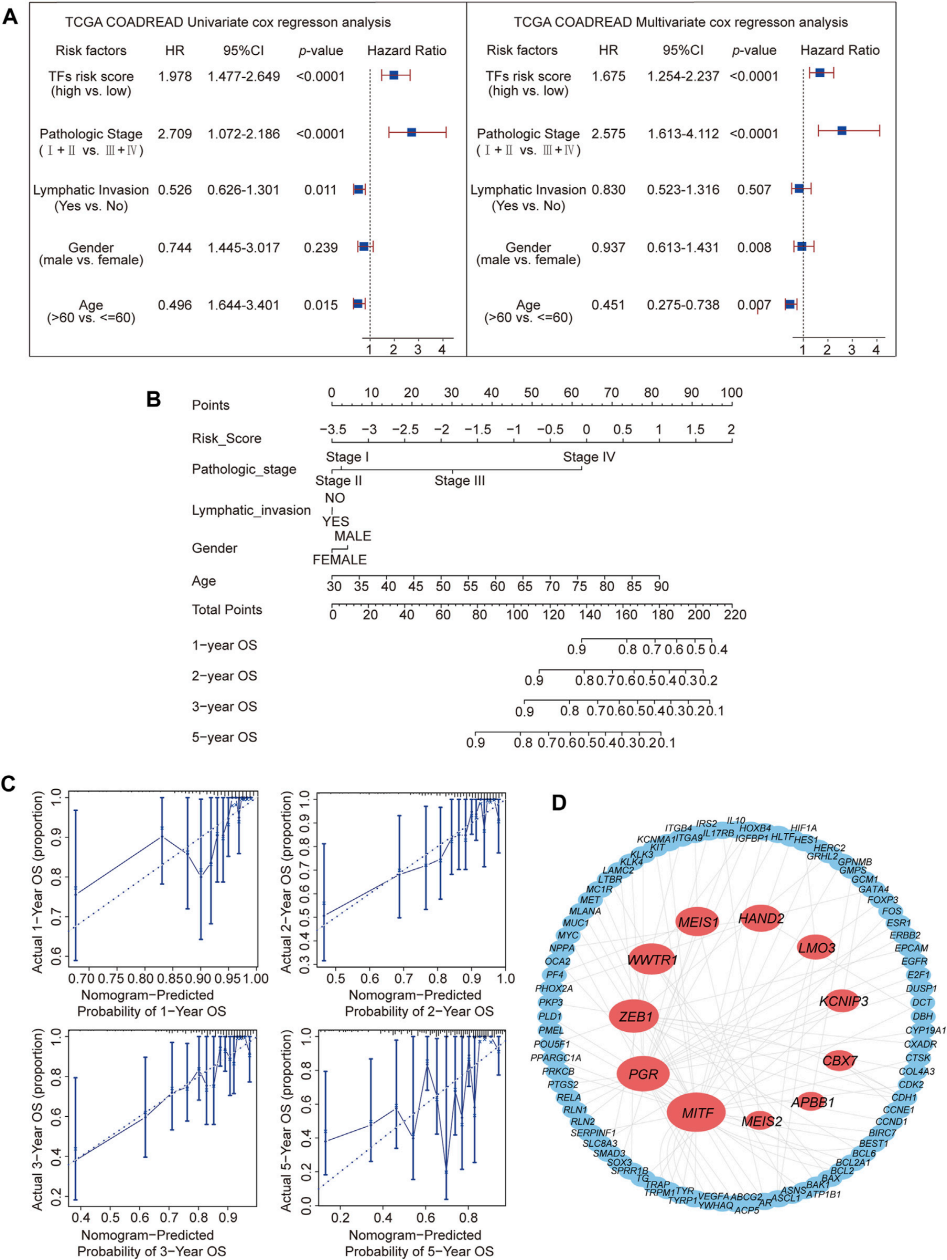
Prediction of the prognosis probability and TF target gene network construction. **(A)** Single-variable Cox regression analysis in the CRC cohort. **(B)** The nomogram for CRC is based on a multivariate Cox regression. **(C)** Calibration curves for 1-, 2-, 3-, and 5-year overall survival. **(D)** Target gene network for 11 TFs.

To explore other additional factors affecting CRC, a total of 317 samples, including phenotypic data, were subjected to Cox single-factor regression analysis for age, sex, pathological stage, lymphatic invasion, and risk score. The analysis revealed that pathological stage emerged as the most influential factor, supported by the hazard ratio, 95% CI, and *p*-value. The multivariate Cox regression analysis showed similar results (Figure 3A). A nomogram was constructed according to the multivariate Cox regression model, which indicated that risk score, pathologic stage, and age contributed significantly to the prognostic analysis, whereas lymphatic invasion and sex exhibited almost no effect (Figure 3B). The calibration curve indicated a higher level of concordance between projected and observed overall survival (OS) rates at 1-year, 2-year, and 3-year intervals compared to the prognostic accuracy for 5-year OS predictions. (Figure 3C).

Typically, TFs modulate downstream genes by binding to their promoters (Yang and Wang, 2021). Therefore, the protein interaction network (details shown in Supplementary Table S4) of the 11 TFs’ targets from the TRRUST database (none of the reported corresponding targets for *NKX3-2* and *TCF7L1*) was analyzed (Figure 3D). This analysis revealed *MITF* with the highest node degree (Yang and Wang, 2021), followed by *PGR* (Langfelder and Horvath, 2008) and *ZEB1* (Lambert et al., 2018). These 3 TFs had high correlation coefficients in the prognostic model. Based on the three most significant TFs identified through LASSO regression analysis, *ZEB1*, *MEIS1*, *MITF*, and *PGR* were identified as the TFs most relevant to CRC prognosis.

### 3.4 Survival analysis and validation of the hub TF functions in CRC

**FIGURE 4:**
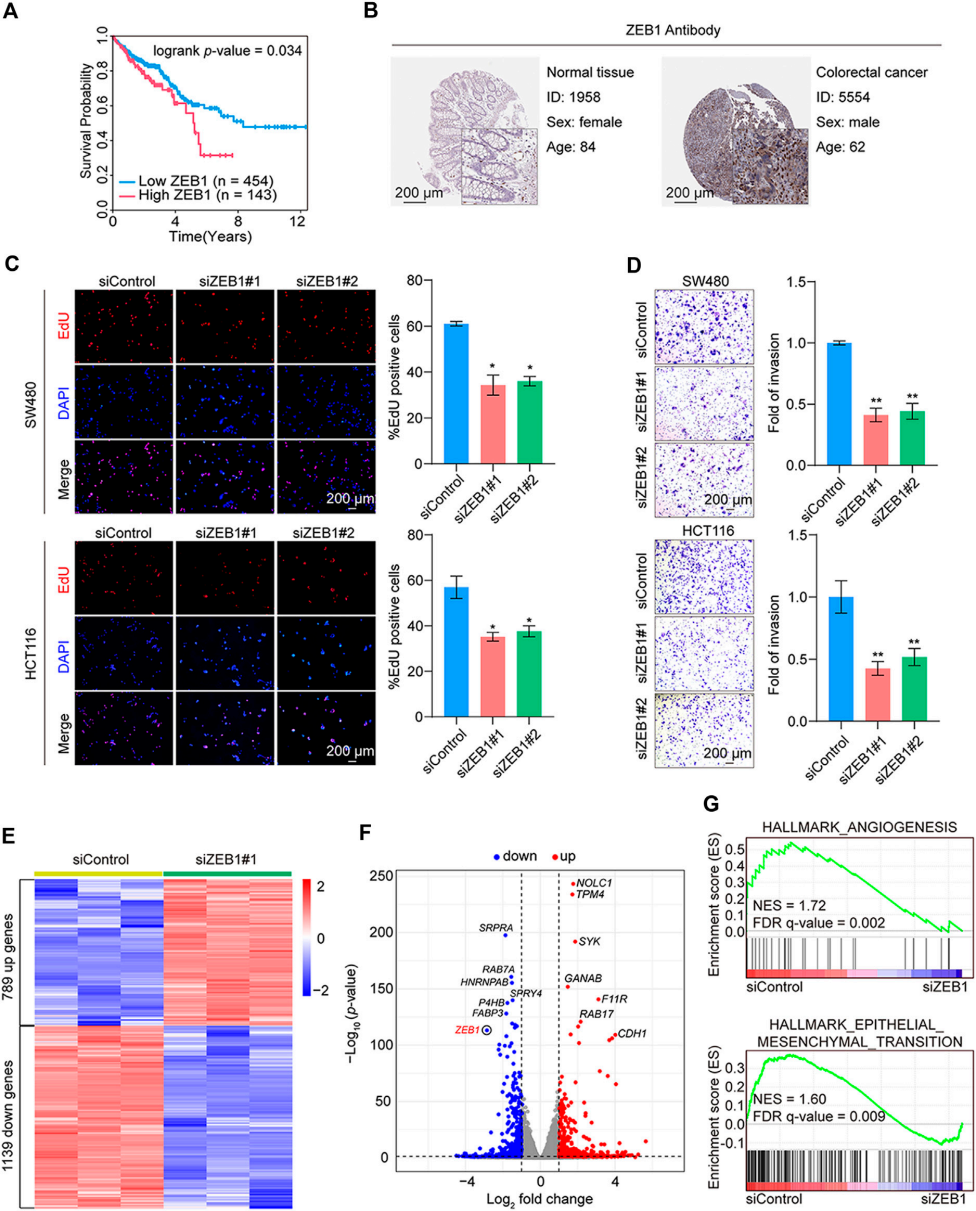
Validation of the hub TF and functional tests in CRC. **(A)** Kaplan-Meier analysis of the hub TF *ZEB1* with overall survival using the online database (https://www.proteinatlas.org/) for CRC. **(B)** Immunohistochemistry of ZEB1 in colorectal cancer and normal samples from the Human Protein Atlas database. **(C)** EdU cell proliferation assay of SW480 and HCT116 cells transfected with siControl or siRNAs targeting ZEB1. Error bars indicate means ± SD. The data was analyzed by two-tailed unpaired *t*-test, *p-value < 0.05 and **p-value < 0.01. **(D)** Transwell assays of SW480 and HCT116 cells transfected with siControl or siZEB1. The images represent one microscopic field in each group. Error bars indicate means ± SD. The data was analyzed by two-tailed unpaired *t*-test, *p-value < 0.05 and **p-value < 0.01. **(E)** Heatmap representation of DEGs (fold change > 1.5; *p*-value < 0.001) in control and ZEB1-knockdown SW480 cells. **(F)** Volcano plot representation of DEGs. **(G)** GSEA of angiogenesis and epithelial-mesenchymal transition pathways. NES, normalized enrichment score; FDR, false discovery rate.

To identify TFs more strongly associated with CRC prognosis, Kaplan-Meier analysis of ZEB1, MITF, PGR, TCF7L1, and MEIS1 was performed. Elevated ZEB1, PGR, and MEIS1 expression were observed to be linked with inferior overall survival outcomes in individuals diagnosed with CRC (https://www.proteinatlas.org/) (Figure 4A; Supplementary Figure S3A). Nevertheless, immunohistochemical results from the Human Protein Atlas database indicated that only ZEB1 protein expression was notably higher in CRC than in the corresponding adjacent tissues (Figure 4B; Supplementary Figure S3B). Based on the outcomes of the TF target gene network analysis, ZEB1 emerged as the most relevant TF for CRC prognosis.

Subsequent results from EdU assay revealed that ZEB1 knockdown suppressed cell proliferation (Figure 4C). The validation of siRNA efficiency in the two CRC cell lines is shown in Supplementary Figures S3C, D. In addition, the Transwell assays showed a marked reduction in cell invasion post-*ZEB1* knockdown in CRC cells (Figure 4D).

Further analysis involved RNA-seq analysis on *in vitro* cultured cells. We employed siRNAs to silence the expression of *ZEB1* in SW480 cells. Three independent controls and three experimental groups targeting *ZEB1* knockdown were employed. Whole-transcriptome clustering analysis identified 789 upregulated and 1139 downregulated genes in the si*ZEB1* group [|fold change| > 1.5, *p*-value < 0.05]. Figures 4E, F depict a heatmap and volcano plot illustrating the DEGs. The DEGs were subjected to gene set enrichment analysis (GSEA), revealing significant enrichment in various cancer-related cellular processes, including angiogenesis and epithelial-mesenchymal transition pathways (Figure 4G). These compelling findings strongly support the notion that ZEB1 plays a pivotal role in promoting the proliferation and invasion of CRC, thereby contributing to cancer progression.

### 3.5 Biological functional analysis of ZEB1

**FIGURE 5:**
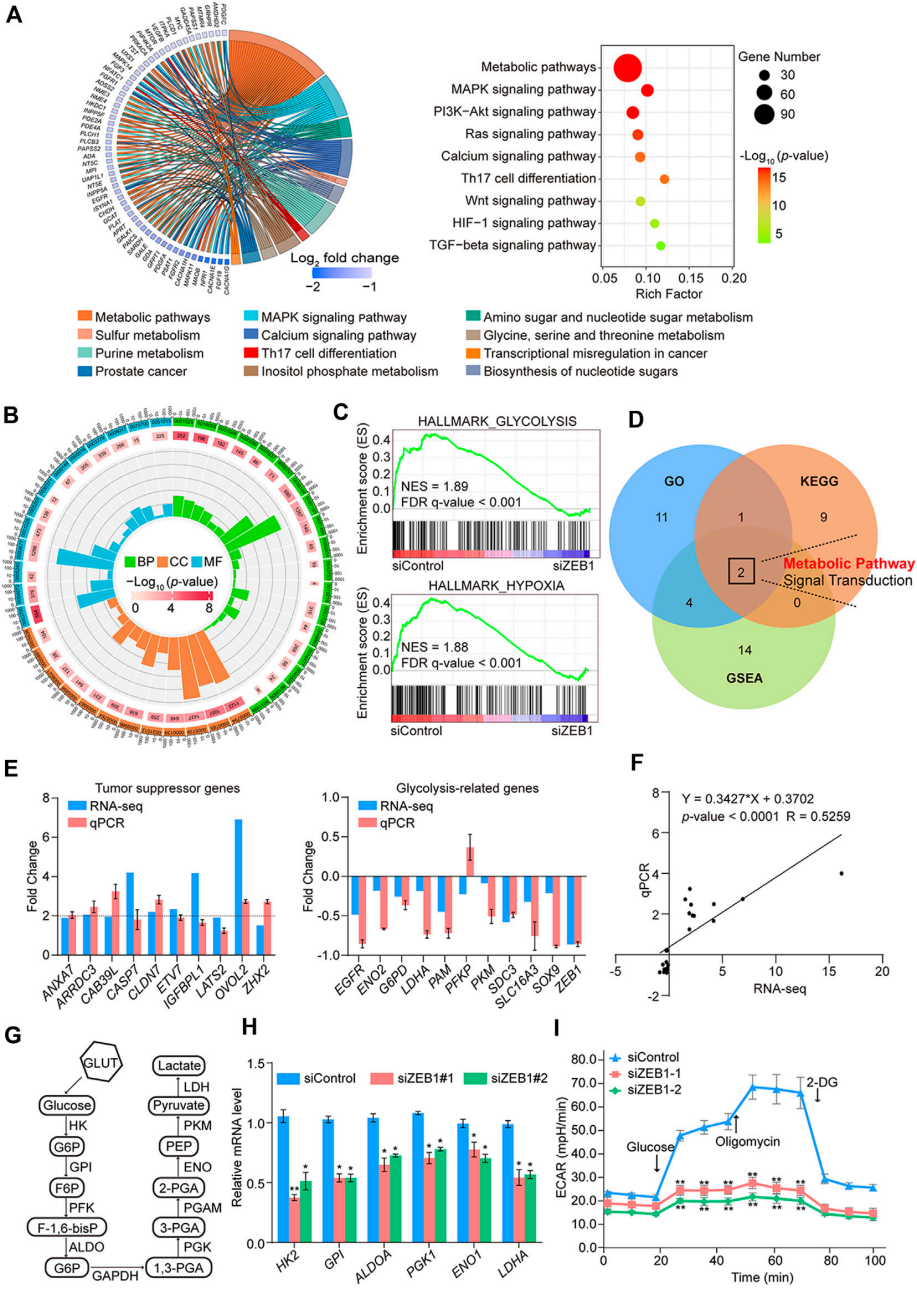
RNA-seq analysis and biological functional analysis of ZEB1 in SW480 cells. **(A)** Results of the KEGG pathway analysis of DEGs in control cells and ZEB1-knockdown cells. Data were analyzed using KOBAS 3.0 software. **(B)** GO enrichment analysis of DEGs in control cells and ZEB1-knockdown cells. **(C)** GSEA analysis plot of the glycolysis pathway and hypoxia pathway. NES, normalized enrichment score; FDR, false discovery rate. **(D)** Venn diagrams of overlapping pathways from GO, KEGG, and GSEA. Comparison of TSG and GRG expression in SW480 cells by column **(E)** and linear fit **(F)** of qRT-PCR vs. RNA-seq. **(G)** Schematic diagram of the glycolysis process. **(H)** qRT-PCR analysis of key enzyme mRNA in the glycolysis pathway in the ZEB1-knockdown SW480 cells. **(I)** Results of ECAR experiments in control cells and ZEB1-knockdown SW480 cells. **(E, H, I)** Error bars indicate means ± SD. The data was analyzed by two-tailed unpaired *t*-test, *p-value < 0.05 and **p-value < 0.01.

To further validate the involvement of ZEB1 in the modulation of CRC with malignant characteristics, our investigation was centered on analyzing RNA-seq outcomes. The KEGG pathway analysis revealed enrichment in metabolic, MAPK signaling, and PI3K-Akt signaling pathways (Figure 5A). Pathways such as cell migration, regulation of glucose metabolic processes, and response to hypoxia were examined using GO analysis (Figure 5B). Interestingly, GSEA analysis revealed significant enrichment in glycolysis and hypoxia pathways in the siControl group but not in the si*ZEB1* group (Figure 5C). These RNA-seq data suggested that contribution of ZEB1 to colorectal tumor growth occurred by coordinating cell metabolism under hypoxic conditions (Figure 5D).

In line with the RNA-seq findings, upregulation of putative tumor suppressor genes (TSGs), such as *ANXA7*, *ARRDC3*, *CAB39L*, *CASP7*, *CLDN7*, *ETV7*, *IGFBPL1*, *LATS2*, *OVOL2*, and *ZHX2*, was observed, underscoring the pivotal role played by epigenetic TSG silencing in driving tumorigenesis (Lee and Muller, 2010; Guo et al., 2023). In contrast, the expression of potential oncogenes, which also belong to glycolysis-related genes (GRGs), including *EGFR*, *ENO2*, *G6PD*, *LDHA*, *PAM*, *PKM*, *SDC3*, *SLC16A3*, and *SOX9*, was diminished in ZEB1-depleted cells (Figures 5E, F). Hypoxia-inducible factor 1α (HIF1α) was a key factor regulating glycolysis. The abnormal expression of *HIF1A* promotes the glycolysis process, including in cancer cells (Semenza, 2003). In light of this information, the enrichment of GRGs indicates a potential significant role for ZEB1 in glucose metabolism. To validate this assumption, we quantified the mRNA levels of GRGs after ZEB1 knockdown. Figure 5G illustrates the key enzymes involved in glycolysis. These findings indicate that ZEB1 promoted the expression of GRGs (Figure 5H) and had a positive impact on glycolysis. Additionally, SW480 cells were transfected with siRNAs, and glycolytic activity was assessed using a Seahorse XFe24 system, revealing that diminished ZEB1 protein levels significantly reduced the ECAR of cells (Figure 5I). This result reflects diminished overall glycolysis levels. Based on these results, it can be speculated that ZEB1 modulates the progression of CRC by regulating certain TSGs and glycolytic processes.

### 3.6 ZEB1 transcriptionally represses TSGs through its interaction with the NuRD complex

**FIGURE 6:**
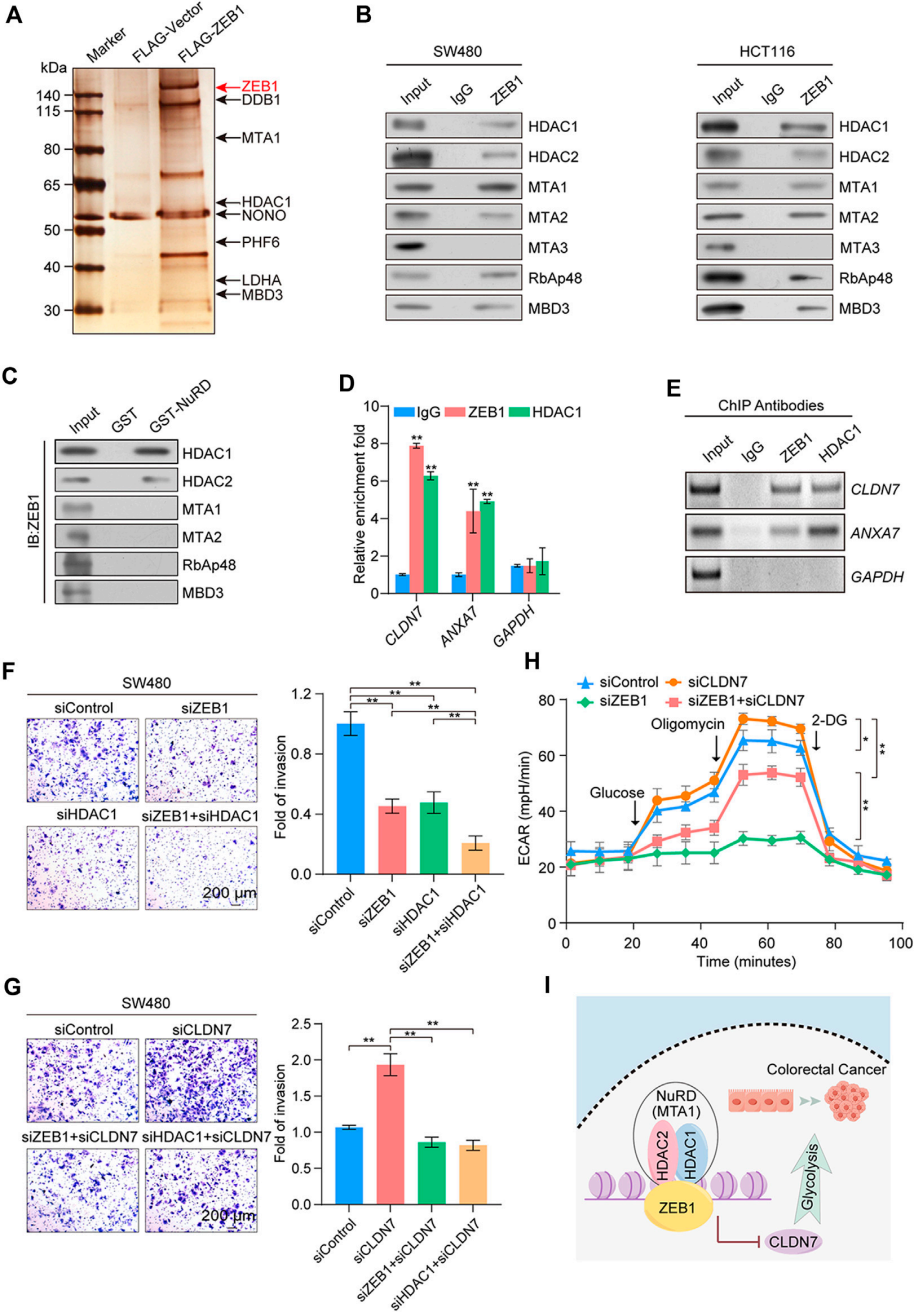
Identification of genome-wide transcription targets for ZEB1. **(A)** Immunoaffinity purification and mass spectrometry analysis of ZEB1-associated proteins in HEK-293T cells. ZEB1 protein bands were retrieved and analyzed using mass spectrometry. **(B)** Association of ZEB1 and the NuRD complex in SW480 and HCT116 cells. Whole-cell lysates were prepared, and coimmunoprecipitation was performed. **(C)** GST pull-down assays with GST-fused NuRD proteins and *in vitro* transcribed/translated ZEB1. **(D)** qChIP analysis of potential ZEB1 target genes in SW480 cells. Results are represented as fold change over control, with GAPDH as the negative control. **(E)** ChIP-PCR analysis in SW480 cells with indicated antibodies. **(F)**, **(G)** Transwell invasion assays of SW480 cells following transfection with corresponding siRNAs. Invading cells were stained and counted. Images represent one field under microscopy in each group. **(H)** Results of ECAR experiments in SW480 cells transfected with control or specific siRNAs. **(I)** A proposed model for the role of ZEB1 in regulating CRC progression. **(D, F–H)** Error bars indicate means ± SD. The data was analyzed by two-tailed unpaired *t*-test, *p-value < 0.05 and **p-value < 0.01.

Affinity purification and mass spectrometry were employed to enhance the mechanistic comprehension of ZEB1’s role in CRC. Mass spectrometry analysis demonstrated that ZEB1 was co-purified with subunits of the NuRD transcription repression complex, including HDAC1, MTA1, and MBD3 (Figure 6A). Supplementary Table S5 provides detailed information on the mass spectrometry results. The interaction between ZEB1 and components of the NuRD complex was validated through Western blotting using antibodies against these specific components in two CRC cell lines (Figure 6B). In addition, GST pull-down assays demonstrated a direct interaction between ZEB1 and HDAC1 as well as HDAC2 (Figure 6C; Supplementary Figure S4A). Notably, although MTA3 is a member of the NuRD complex, it did not interact with the ZEB1/NuRD complex.

Because of the preferential binding of ZEB1 with HDAC1 over HDAC2, we next performed ChIP experiments with specific antibodies against ZEB1 and HDAC1. This analysis was aimed at exploring the mechanism by which the ZEB1/NuRD(MTA1) complex co-regulates the CRC process. qChIP analysis was employed for *ANXA7*, *ARRDC3*, *CAB39L*, *CASP7*, *CLDN7*, *ETV7*, *IGFBPL1*, *LATS2*, *OVOL2*, and *ZHX2*. The ZEB1/NuRD complex was significantly enriched in the *CLDN7*, *ANXA7*, *CAB39L*, and *ETV7* promoter regions (Figure 6D; Supplementary Figure S4B). Due to the absence of substantial disparities in the protein levels of CAB39L, ETV7, OVOL2, and ZHX2 between neoplastic and neighboring tissues (Supplementary Figure S4C) and the lack of known immunohistochemical results for ARRDC3 in CRC, ChIP-PCR analyses were employed for *ANXA7* and *CLDN7* (Figure 6E). The results demonstrated a robust enrichment of ZEB1 and HDAC1 on the promoters of *CLDN7* and *ANXA7*, all of which are implicated in tumor suppression. The mRNA and protein levels of CLDN7 and ANXA7 in ZEB1- or HDAC1-knockdown SW480 cells were significantly increased (Supplementary Figure S4D). To gain further insights into the precise molecular mechanisms underlying ZEB1’s regulation of CRC, we conducted an extensive analysis using publicly available clinical datasets from the TCGA and GEO databases (GSE100179). This analysis demonstrated a negative correlation between CLDN7 expression and ZEB1, while ANXA7 showed the opposite trend (Supplementary Figure S4E). Moreover, Kaplan-Meier survival analysis demonstrated a significant correlation between increased CLDN7 expression and improved overall survival rates among patients with CRC (Supplementary Figure S4F).

The co-knockdown of ZEB1 and HDAC1 enhanced the effect of the knockdown of ZEB1 or HDAC1 alone (Figure 6F), whereas the knockdown of CLDN7 significantly restored the cell invasion ability, which was diminished by the knockdown of ZEB1 or HDAC1 alone (Figure 6G). Notably, the decrease in cellular glycolysis induced by ZEB1 deficiency was reversed by CLDN7 knockdown (Figure 6H). The expression of indicated proteins was measured by Western blotting (Supplementary Figures S4G, H). In summary, our findings demonstrate that the ZEB1/NuRD complex collaboratively suppressed the transcription of the tumor suppressor gene *CLDN7*, promoted glycolysis, and exerted an impact on tumor development (Figure 6I), potentially signifying the discovery of novel CRC biomarkers.

## 4 Discussion

TFs account for approximately 8% of the total human gene pool and exhibit associations with a diverse range of diseases and phenotype variations (Shi et al., 2016; Lambert et al., 2018), spanning from diabetes (Gonzalez et al., 2018), inflammatory disorders (Ross and Cantrell, 2018), and cardiovascular disease (Papanicolaou et al., 2008) to many cancers (Yu et al., 2009; Stine et al., 2015; Zou et al., 2020; Wu M. J. et al., 2021). Dysregulated TFs play a pivotal role in the pathogenesis of these diseases, underscoring their potential as valuable prognostic markers and therapeutic targets, particularly in cancer. To our knowledge, this study represents the first identification of prognosis-related TFs in CRC using advanced methodologies such as WGCNA and Cox regression analyses. Using WGCNA, we extracted core gene networks and identified biologically relevant modules involving functionally related TFs and attentional phenotypes in CRC. Considering that TFs in the turquoise model were highly correlated with OS_status, further scrutiny using LASSO regression analysis led to the identification of 13 prognostic TFs (*ZEB1*, *TCF7L1*, *MEIS1*, *MITF*, *NKX3-2*, *PGR*, *MEIS2*, *WWTR1*, *KCNIP3*, *APBBB1*, *CBX7*, *HAND2*, and *LMO3*). To isolate the most representative prognostic TFs, we then constructed a regulatory network diagram based on the interactions among these 13 TFs and performed survival analyses and phenotypic experiments using the top five hub TFs (*ZEB1*, *TCF7L1*, *MEIS1*, *MITF*, and *PGR*). This analysis finally pointed to *ZEB1* as the most closely associated TF with CRC. Subsequently, we conducted a comparative between the results of the WGCNA and COX regression analyses with the actual expression of tumor cells, validating the prognostic model and establishing the hub gene *ZEB1* as a CRC prognostic factor.

Although previous research applied WGCNA and Cox regression analysis to assessing tumors, none exclusively focused on identifying prognosis-related TFs in CRC. For example, Zhai et al. (2017) revealed five recurrence-associated molecular and prognostic indicators in colon cancer using WGCNA in colon cancer but did not construct a prognostic model using Cox regression analysis. Another study examined the correlations between stemness genes and prognosis in CRC (Wei et al., 2021) using WGCNA and LASSO-penalized Cox regression analyses; however, it did not specifically identify prognosis-related TFs. Because our study focused only on prognosis-related TFs, it is likely to be more precise. Moreover, the establishment of an independent prognostic factor and an effective prognostic model contributes to the credibility of our findings.

ZEB1 is predominantly recognized for its role in driving the epithelial-to-mesenchymal transition (EMT) in cancer cells, a process that promotes tumor progression (Larsen et al., 2016a). The upregulation of *ZEB1* expression has been observed to exhibit a positive correlation with elevated tumor grade and metastasis across various types of cancers (Zheng et al., 2015; Larsen et al., 2016b). Further, ZEB1 plays a pivotal part in shaping the tumor microenvironment and maintaining functions that support macrophages associated with tumors (Cortes et al., 2017; Jiang et al., 2020). Although previous research, such as that by Sun et al. hinted at *ZEB1* regulation by TCF4 contributing to drug resistance and stemness in CRC (Sun et al., 2020), the specific mechanism by which ZEB1 influences CRC prognosis remains unclear.

The NuRD complex exhibits histone deacetylation activity and primarily functions in transcriptional repression programs to regulate cancer metastasis (Liu et al., 2023). ZEB1 recruits the NuRD complex to form a transcriptional inhibitory unit, which was confirmed for the first time by using mass spectrometry analysis in CRC. The expression of *ANXA7*, *CLDN7*, *ETV7*, and *CAB39L* was inhibited by the newly formed complex. Furthermore, the ZEB1/NuRD(MTA1) complex was confirmed to regulate glycolysis and promote invasiveness of cancer cells, suggesting that the NuRD complex plays a role in glycolysis and cancer progression in CRC.

**FIGURE 7:**
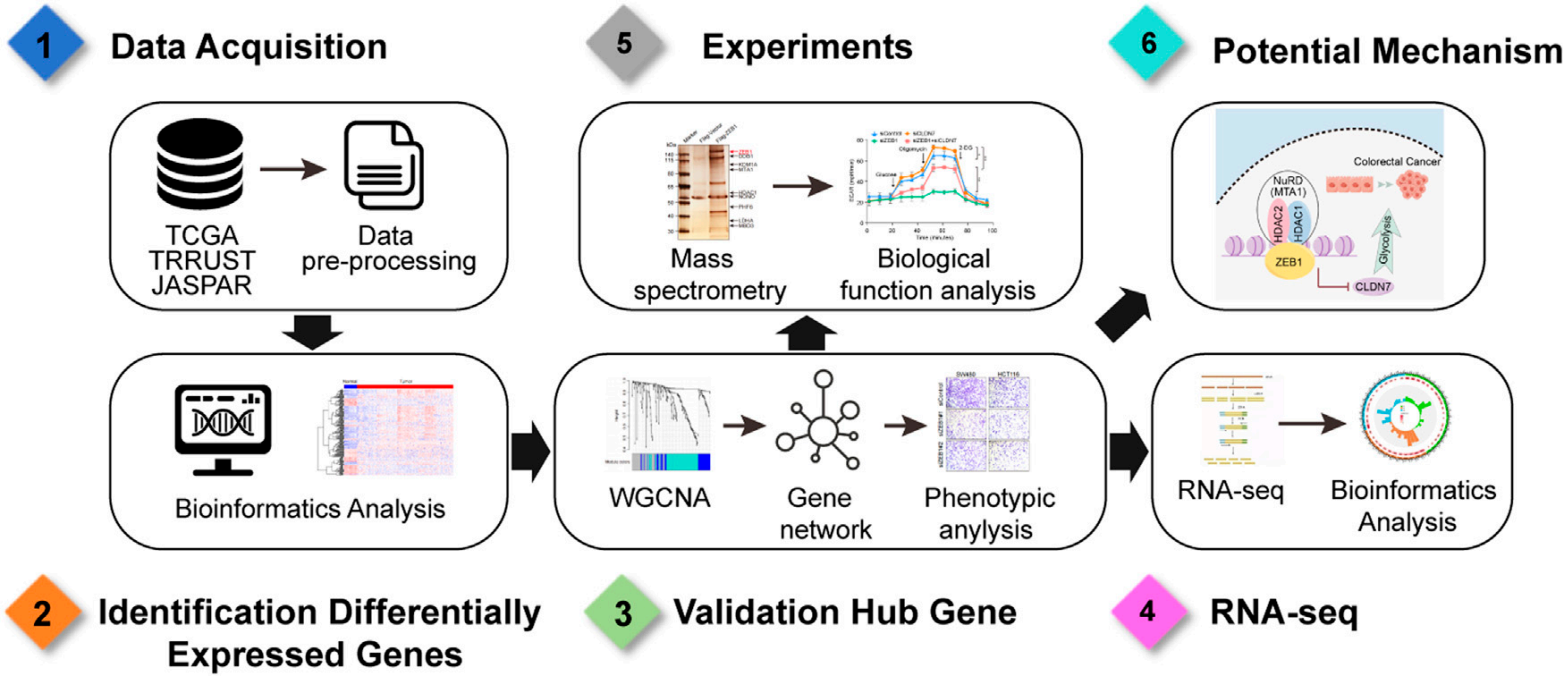
Workflow diagram of analyses.

The Warburg effect refers to the aberrant metabolism of cancer cells, wherein they undergo high glycolysis even in oxygen-rich environments (Zhang et al., 2019; Hou et al., 2020). This modified metabolic process results in epigenetic and genetic modifications, leading to the emergence of numerous novel cellular phenotypes that augment the proliferation and aggressiveness of cancer cells (Li et al., 2018; Nie et al., 2020; Park et al., 2021). While reprogrammed cellular metabolism is a widely accepted hallmark of cancer (Hanahan and Weinberg, 2011), our study adds to this by reporting for the first time that ZEB1 significantly enhances glycolysis in CRC cells. We investigated the specific molecular mechanism underlying this physiological process by using immunoaffinity purification and ChIP techniques. Our results pinpointed a direct interaction between ZEB1 and the NuRD(MTA1) complex that collaboratively suppresses the expression of the TSG *CLDN7*. Previous studies have suggested that a loss of function in TSGs contributes to cancer cell malignancy (Lee and Muller, 2010). Further, Bhat et al. demonstrated that CLDN7 overexpression induces epithelial characteristics and inhibits CRC cells growth (Bhat et al., 2015). Moreover, CLDN7 also plays a crucial role cancer cell carbohydrate metabolism (Ding et al., 2022). However, in recent years, research on CLDN7’s role in regulating cancer glycogen metabolism has been limited. Thus, our findings, along with previous research, establish that the hub TF *ZEB1* promotes glycolysis in CRC cells by inhibiting metabolism-related TSG *CLDN7*, which is not conducive to prognosis (Figure 7). Consequently, ZEB1 could potentially serve as both a diagnostic and prognostic marker due to its multifunctionality in CRC.

In conclusion, our study utilized a comprehensive CRC dataset obtained from the TCGA database to identify TFs exhibiting differential expression patterns between normal and cancerous samples. By employing the WGCNA approach, we categorized functionally related TFs into biologically meaningful modules that were strongly linked to cancer progression and an attentional phenotype in CRC. Using a prognosis-related risk model, we successfully identified 13 prognosis-related TFs. Finally, ZEB1 emerged as a hub TF by combining the regulatory network diagram with the actual expression profiles within tumor cells. Crucially, we identified a clear molecular mechanism through which ZEB1 affects CRC progression and prognosis. Our study provides a direct reference for exploring prognosis-related TFs and is significant for understanding the roles of TFs in CRC. Despite these useful insights, our study has limitations. For example, the screened TFs are not the only factors affecting prognosis, indicating that the prognostic effect of related TFs may be slightly less than that of comprehensive factors at the pathological stage. Further investigations are imperative to elucidate the intricate molecular mechanisms and substantiate the pivotal role of ZEB1 in both diagnosis and therapeutic interventions for CRC.

## 5 Conclusion

In summary, our study has identified ZEB1 as a pivotal biomarker associated with cancer prognosis and elucidated the intricate molecular mechanism by which ZEB1 promotes CRC. The collaborative action of the ZEB1/NuRD complex transcriptionally represses the tumor suppressor gene *CLDN7*, stimulates glycolysis, and facilitates tumor progression. These findings present compelling evidence supporting the consideration of ZEB1 as a robust diagnostic biomarker and a potential therapeutic target for CRC.

## Data Availability

The original data presented in the study is deposited in the Gene Expression Omnibus (GEO) repository, accession number is GSE272912.
