# Supplementary material for: Transcription factor ZEB1 coordinating with NuRD complex to promote oncogenesis through glycolysis in colorectal cancer
Source: Front Pharmacol. 2024 Aug 13;15:1435269. doi: 10.3389/fphar.2024.1435269 (PMC11347313; doi:10.3389/fphar.2024.1435269)

Figure 6B left

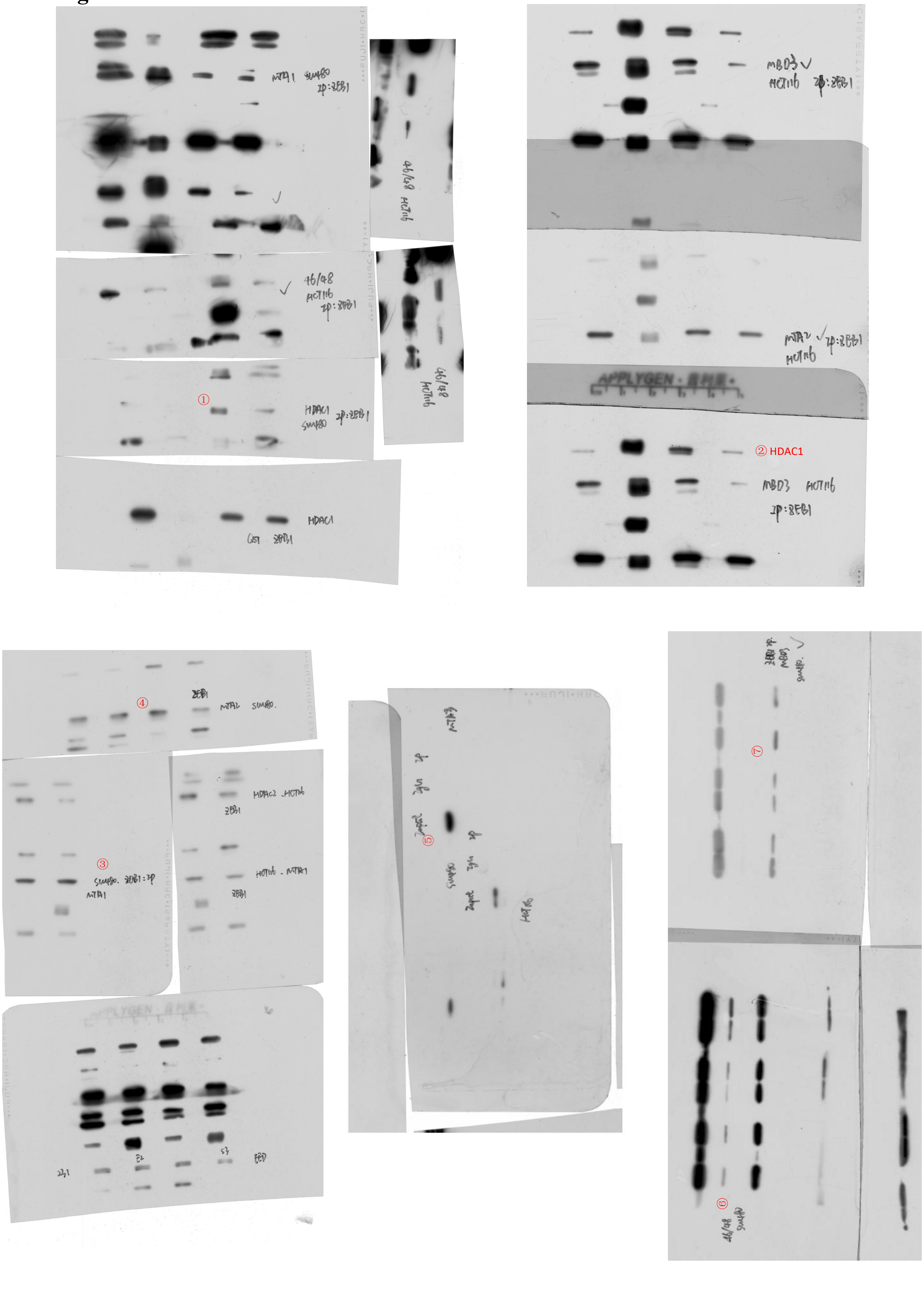

Figure 6B right

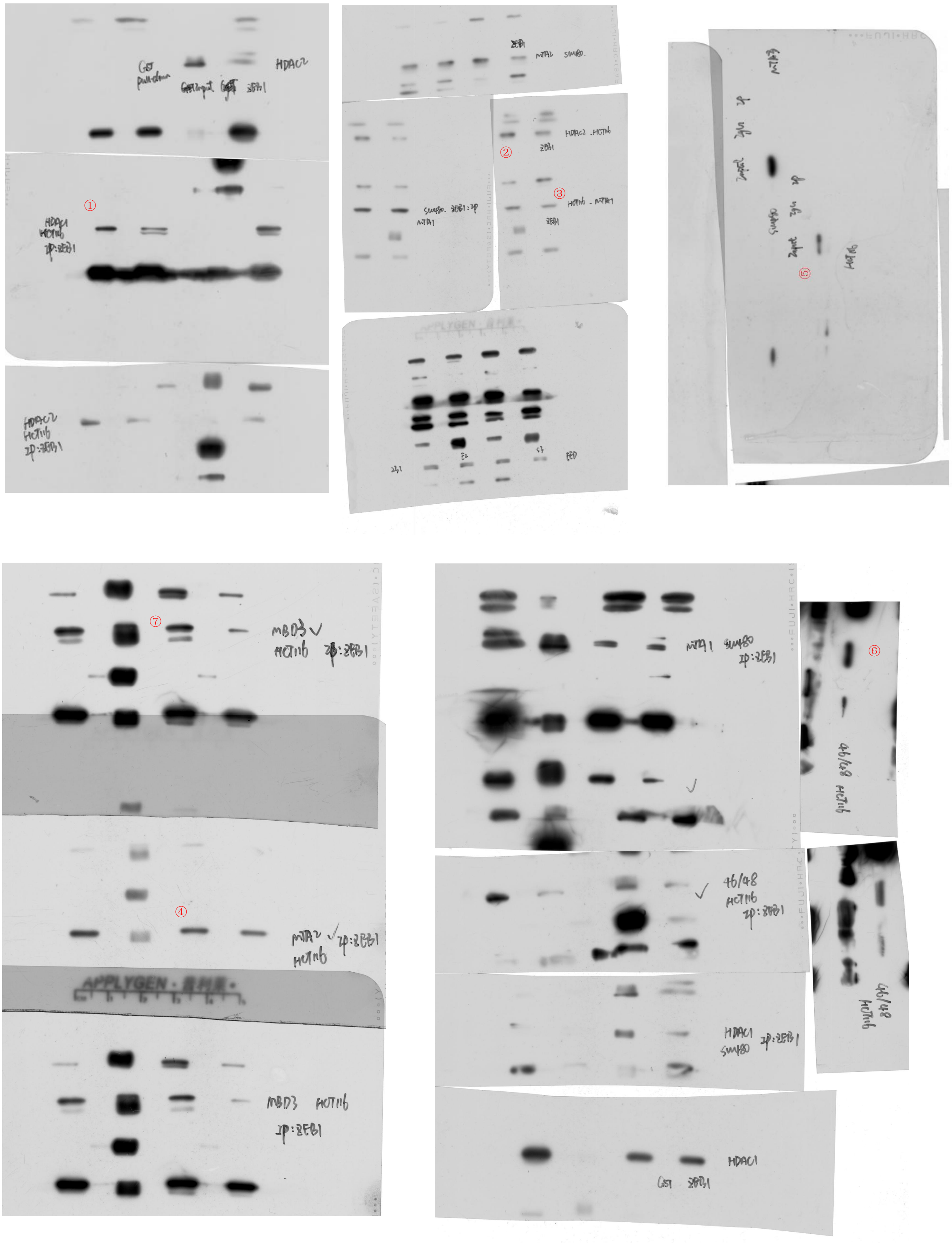

Figure 6C

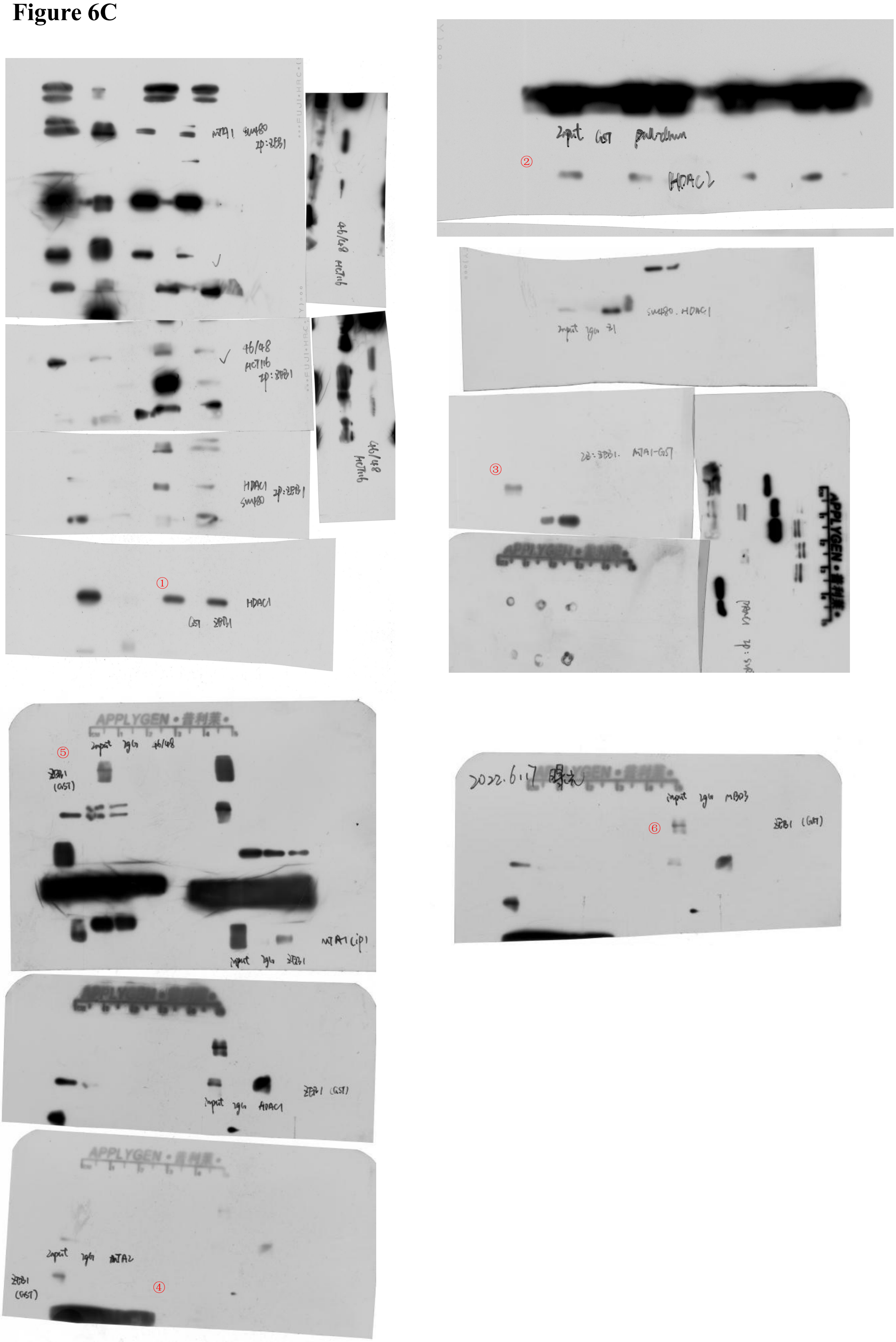

Figure 6E (Agarose gel electrophoresis)

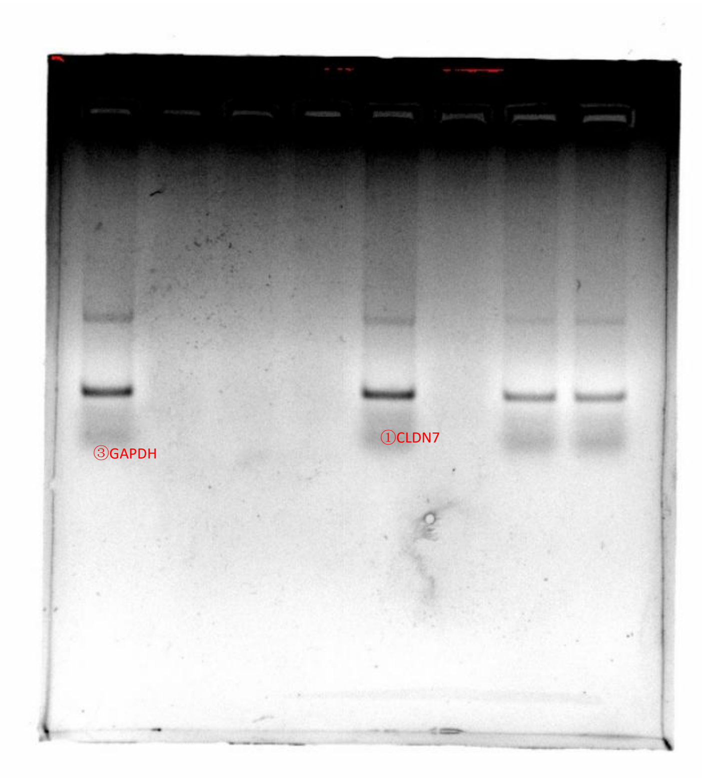

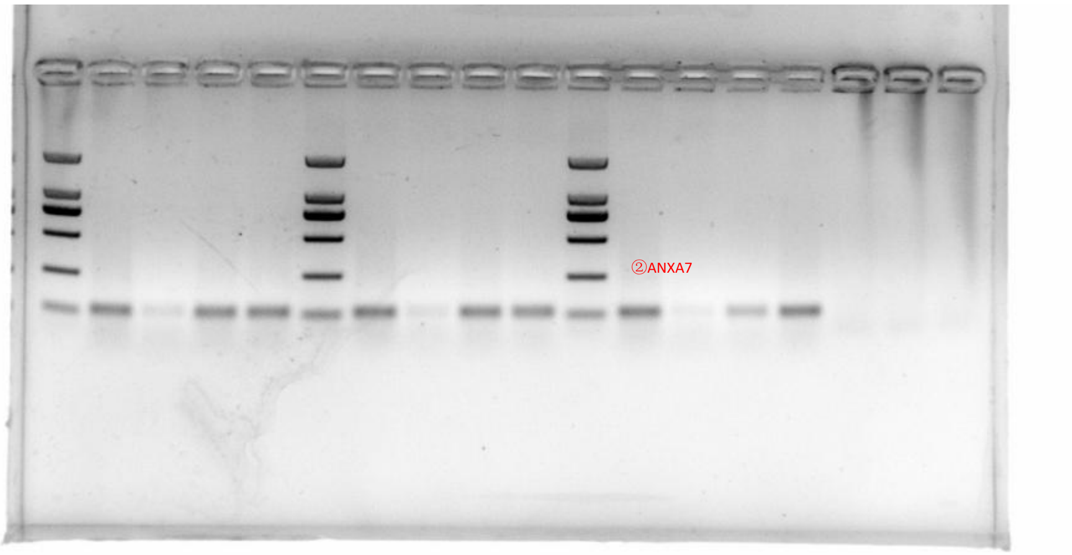

Supplementary Figure S3C

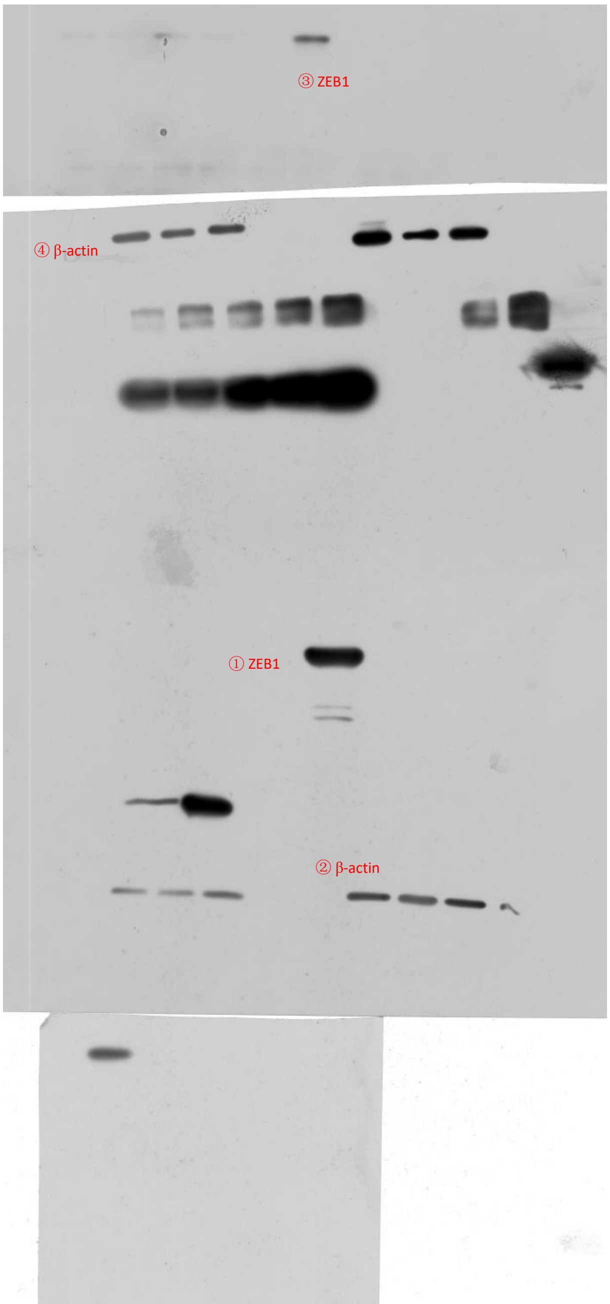

Supplementary Figure S4D

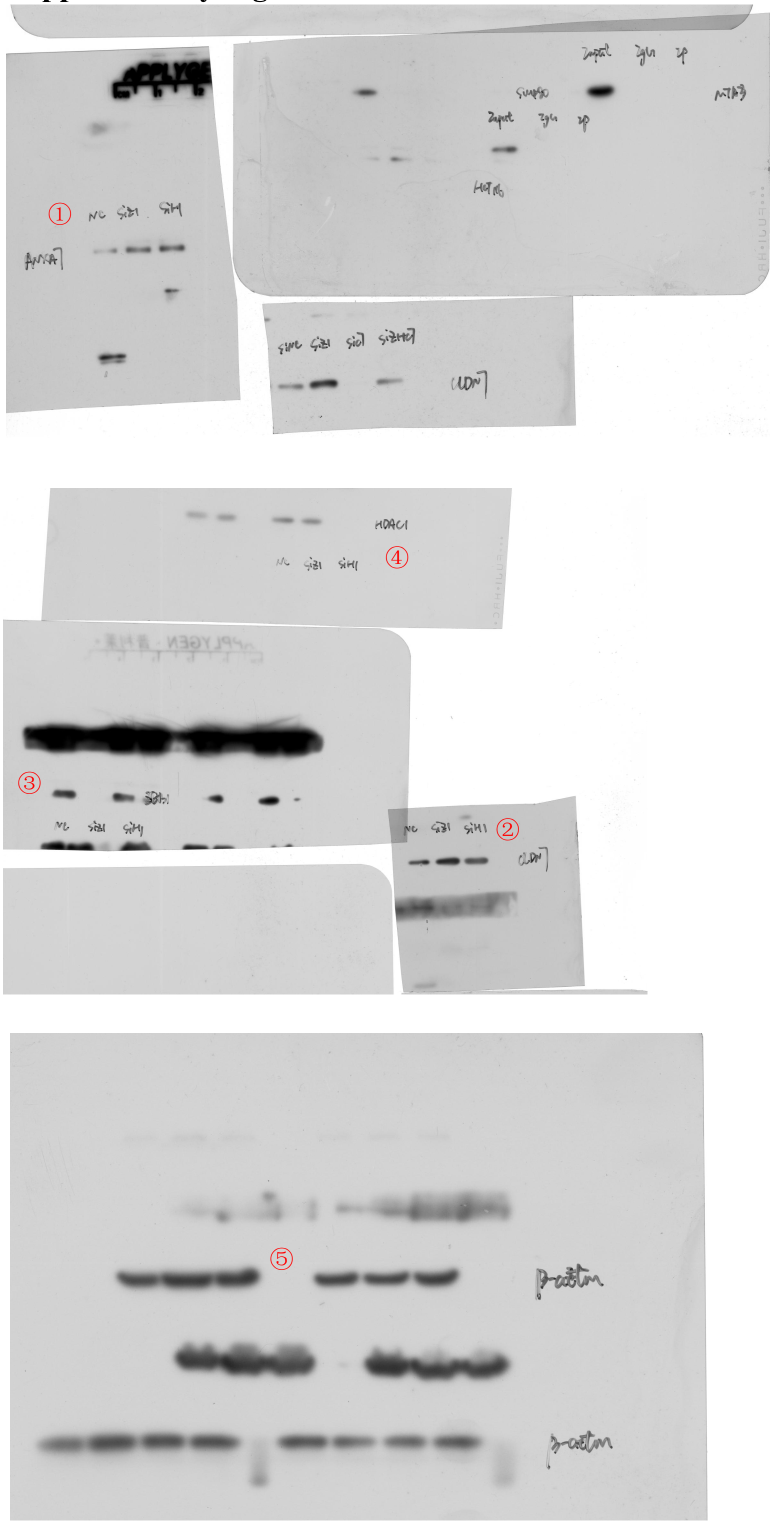

Supplementary Figure S4G

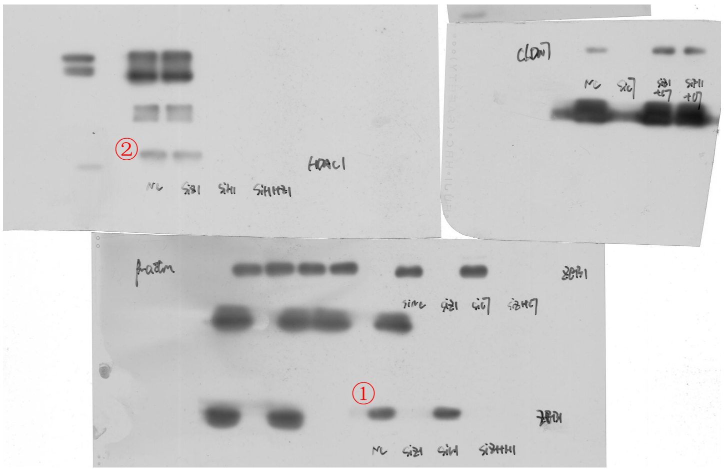

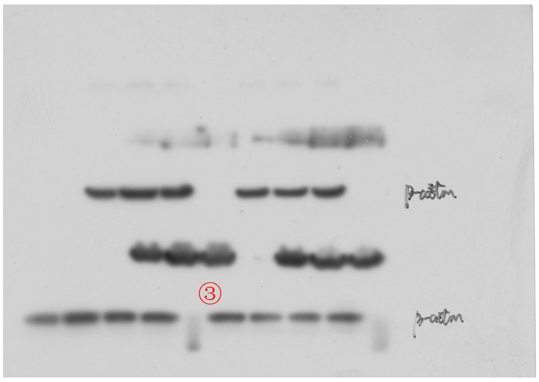

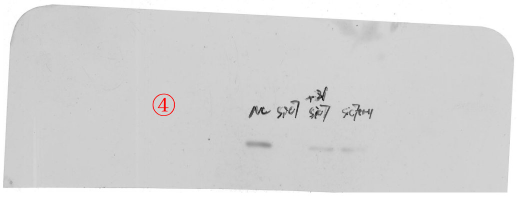

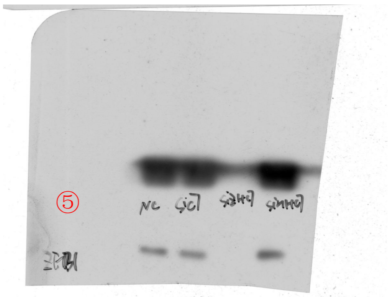

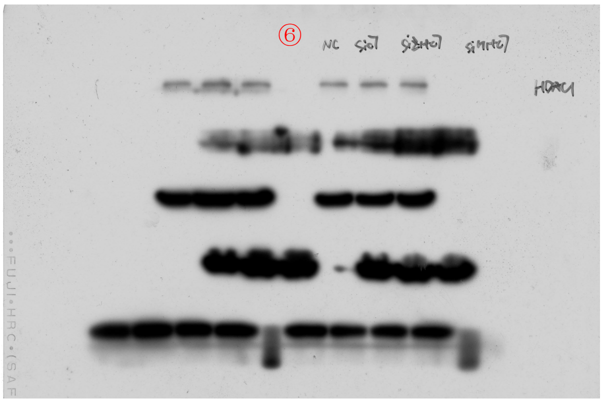

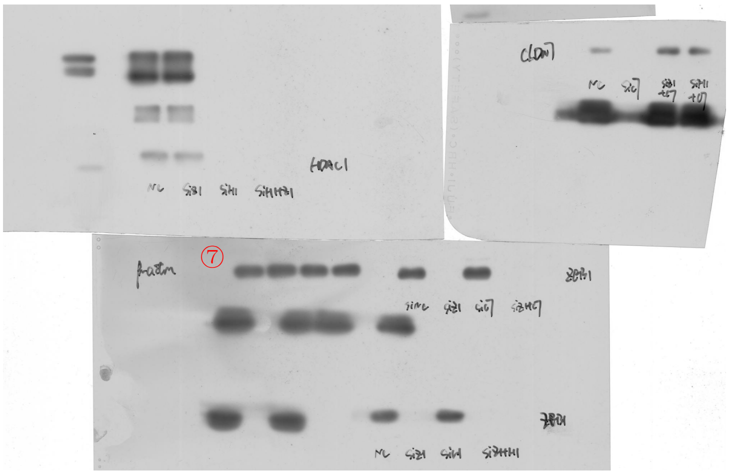

Supplementary Figure S4H

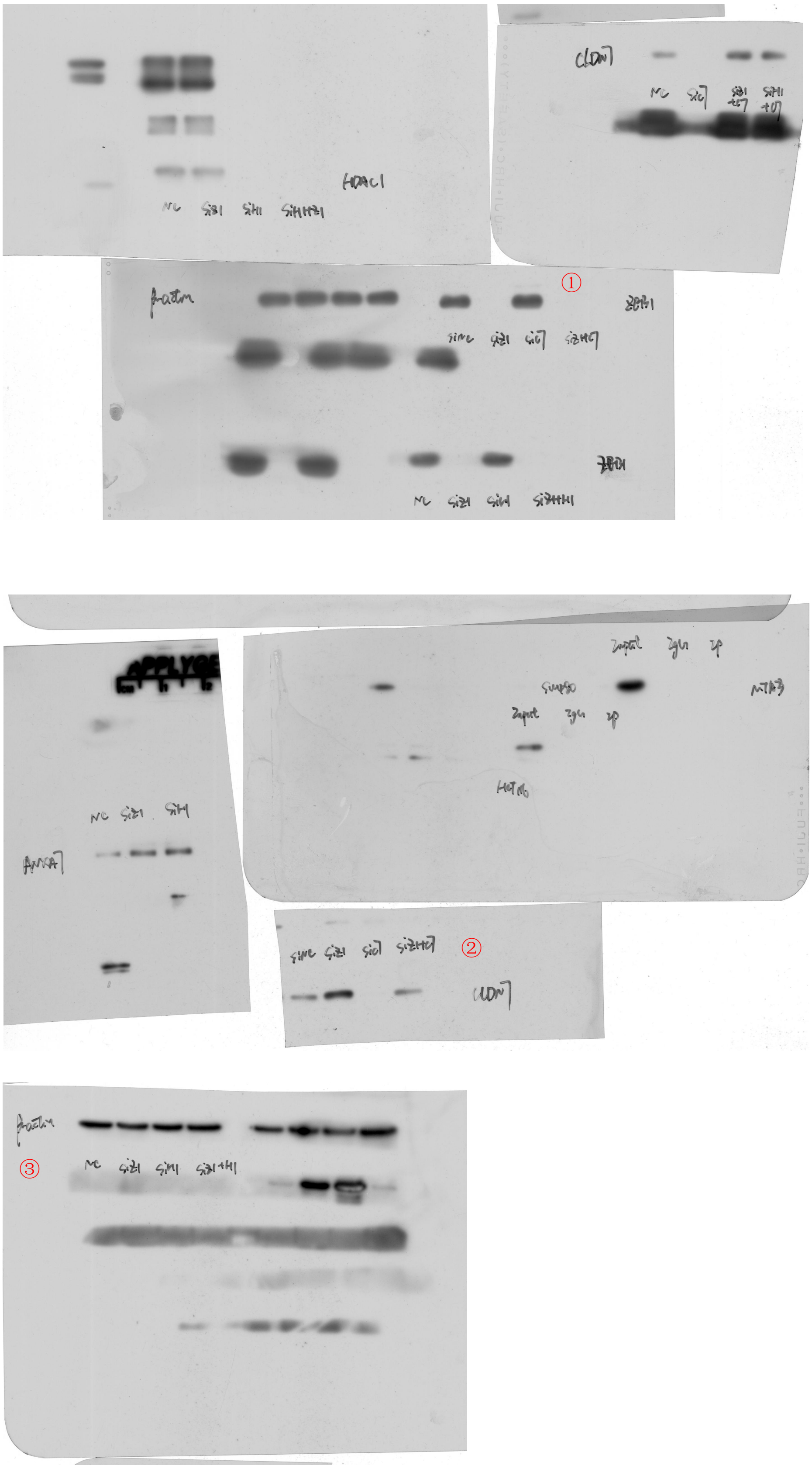

Supplement: Supplementary file 1 [file DataSheet2.pdf]
