# Supplementary material for: Transcription factor ZEB1 coordinating with NuRD complex to promote oncogenesis through glycolysis in colorectal cancer
Source: Front Pharmacol. 2024 Aug 13;15:1435269. doi: 10.3389/fphar.2024.1435269 (PMC11347313; doi:10.3389/fphar.2024.1435269)
Supplement: Supplementary file 3 [file DataSheet1.docx]

Supplementary Figures


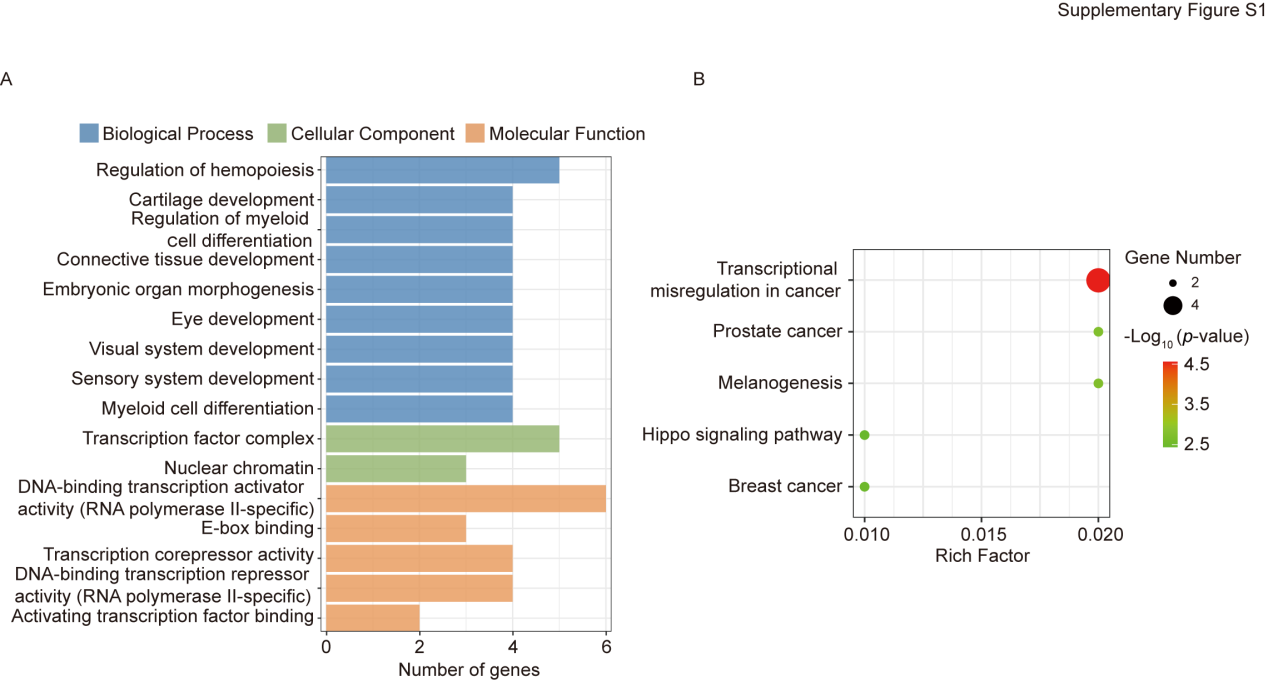


Supplementary Figure S1

Functional enrichment analysis bubble chart of differently expressed TFs. GO enrichment analysis of differently expressed TFs **(A)** and KEGG enrichment analysis of differentially expressed TFs **(B)**.


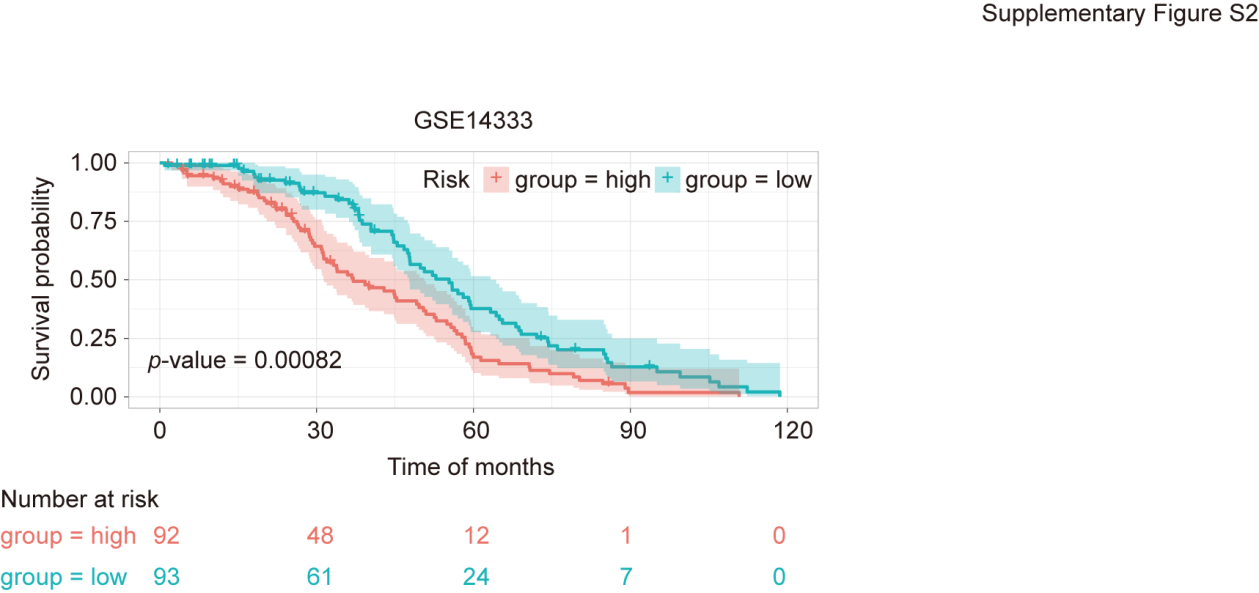


Supplementary Figure S2

Kaplan-Meier curve diagram of overall survival of high and low risk groups from an external data set validating the prognostic risk score model.


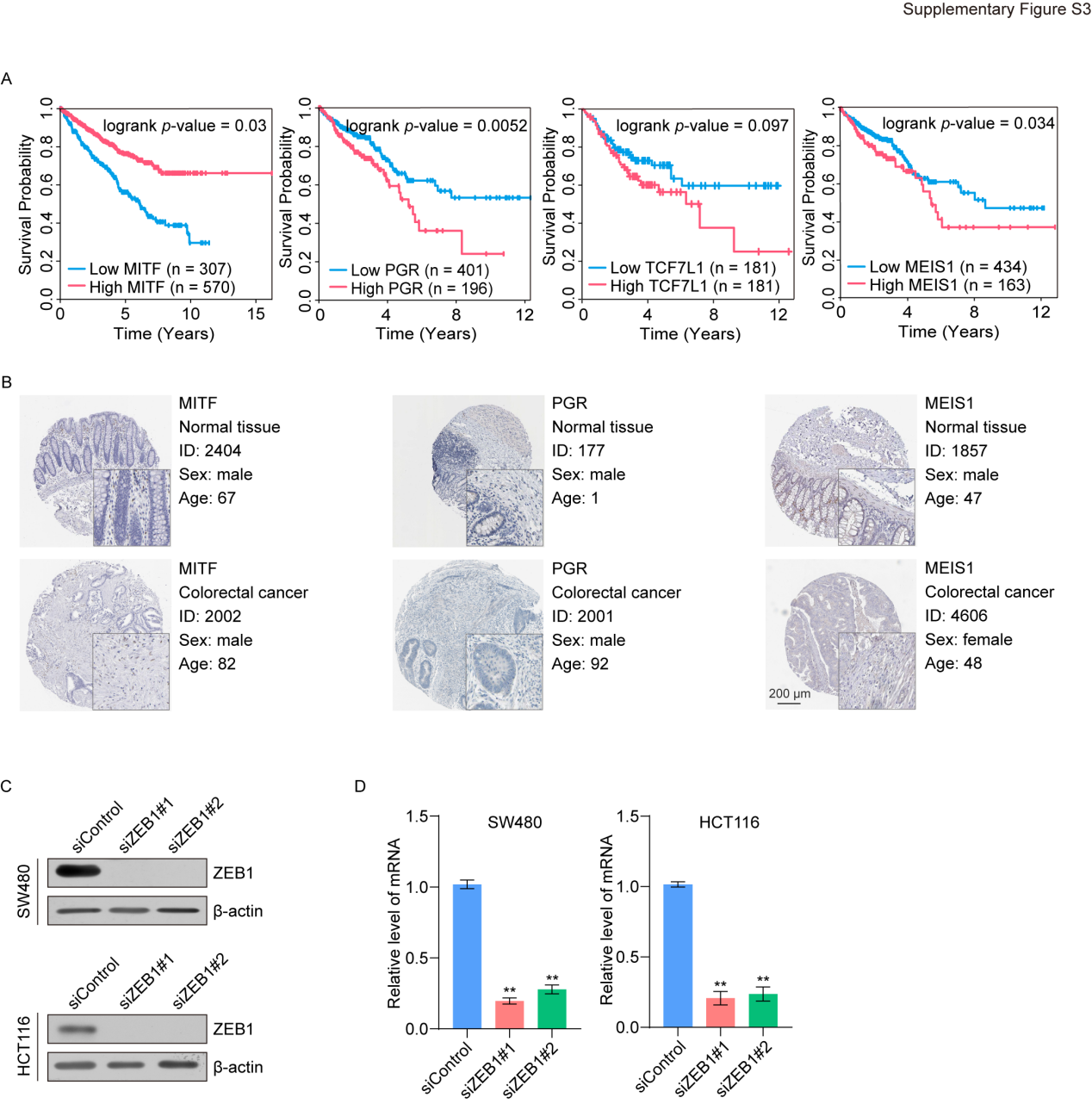


Supplementary Figure S3

**(A)** Kaplan-Meier analysis of four TFs (MITF, PGR, TCF7L1, and MEIS1) with overall survival in the online database (https://www.proteinatlas.org/) in the CRC. **(B)** IHC of MITF, PGR, and MEIS1, in colorectal cancer and normal samples from the HPA database. **(C)**-**(D)** The validation of ZEB1 siRNA efficiency in SW480 and HCT116 cell lines. Error bars indicate means ± SD. The data was analyzed by two-tailed unpaired *t*-test, **p*-value < 0.05 and ***p*-value < 0.01.


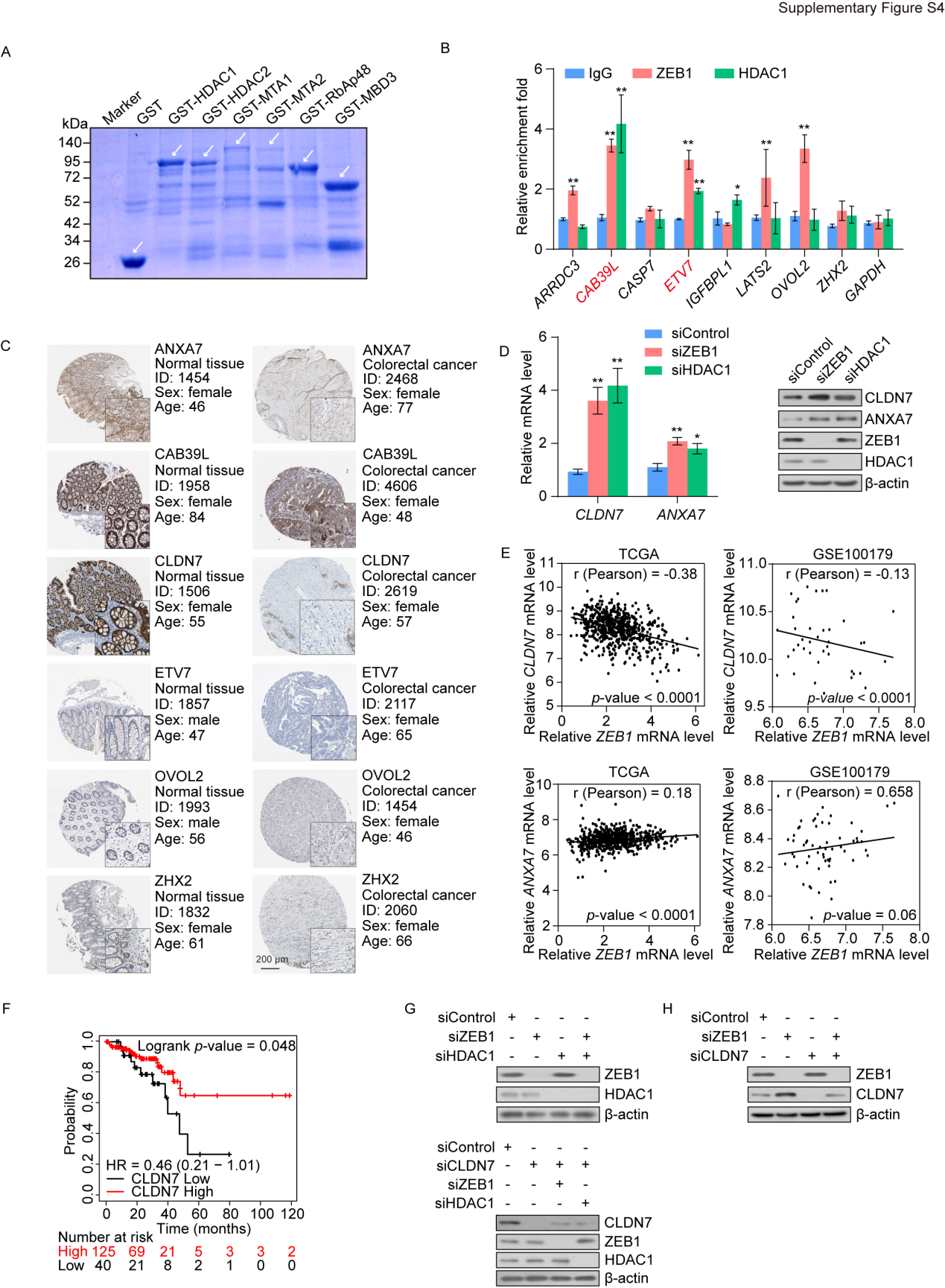


Supplementary Figure S4

**(A)** GST-fused NuRD proteins purified from BL21 *Escherichia coli*. (used in Figure 6C). **(B)** qChIP analysis of potential ZEB1 and HDAC1 target genes in SW480 cells. Results are represented as fold change over control, with GAPDH as a negative control. **(C)** IHC analysis of ANXA7, CAB39L, CLDN7, ETV7, OVOL2, and ZHX2 in colorectal cancer and normal samples from the HPA database. **(D)** qRT-PCR and western blot analysis indicated proteins in SW480 cells transfected with control siRNAs or siRNAs targeting ZEB1 and HDAC1. **(E)** Analysis of public datasets (TCGA and GSE100179) from colorectal cancer for the expression of ZEB1, CLDN7, or ANXA7 was performed using a two-tailed unpaired *t*-test. **(F)** Kaplan-Meier survival analysis of overall survival of CLDN7 in colorectal tissue performed using the online tool Kaplan-Meier plotter (http://kmplot.com/analysis/). **(G)** Western blot analysis of indicated proteins in the ZEB1/HDAC1-knockdown or ZEB1/CLDN7/ HDAC1-knockdown SW480 cells. **(H)** Western blot analysis of indicated genes in the ZEB1-knockdown and CLDN7-knockdown SW480 cells. (B, D) Error bars indicate means ± SD. The data was analyzed by two-tailed unpaired *t*-test, **p*-value < 0.05 and ***p*-value < 0.01.
